# Evolution of the Aroma of Treixadura Wines during Bottle Aging

**DOI:** 10.3390/foods9101419

**Published:** 2020-10-08

**Authors:** Iván Vázquez-Pateiro, Uxía Arias-González, José Manuel Mirás-Avalos, Elena Falqué

**Affiliations:** 1 Depto. Química Analítica, Facultad de Ciencias, Universidade de Vigo, As Lagoas s/n, 32004 Ourense, Spain; ivpateiro@yahoo.es (I.V.-P.); uxiaariasgonzalez@gmail.com (U.A.-G.); 2 Unidad de Suelos y Riegos (Asociada a EEAD-CSIC), Centro de Investigación y Tecnología Agroalimentaria de Aragón (CITA), Avda. Montañana 930, 50059 Zaragoza, Spain; 3 Clúster de Investigación y Transferencia Agroalimentaria del Campus Auga (CITACA), As Lagoas s/n, 32004 Ourense, Spain

**Keywords:** bottle aging, flavor profile, sensory evaluation, volatile composition, white wine

## Abstract

Aroma is a crucial attribute for wine quality, particularly in white wines. Traditionally, the consumption of young white wines is recommended over the year following grape harvest due to potential aroma losses that would worsen wine quality. This study aimed to investigate the evolution of volatile compounds, odor activity value-based aroma notes, and sensory perception in Treixadura (*Vitis vinifera* L.) dry white wines during a 24-month bottle-aging period. Volatile composition was determined by gas chromatography, and wine sensory evaluation was performed by experts. Wine samples had similar volatile compositions at the time of bottling. The volatile contents of the wines were respectively 322.9, 302.7, 323.0, and 280.9 mg L^−1^ after 6, 12, 18, and 24 months of bottle storage. Most of the volatiles tended to maintain constant concentrations, or with slight increases in all families of volatiles except for acetates and carbonyl compounds, until two years after harvest (18 months of bottle storage) and, then, concentrations reduced sharply. After 24 months of storage in the bottle, the concentrations of terpenes, C6 compounds, higher alcohols, ethyl esters, fatty acids, acetates, carbonyl compounds, and volatile phenols were reduced by 32%, 47%, 11%, 39%, 50%, 74%, 41%, and 54%, respectively. The 18-month bottle-aged wines showed the highest concentrations of volatiles, as well as the best performance in the sensory evaluation, suggesting that a good balance of the aroma attributes was achieved on this date. In conclusion, the current study suggests that Treixadura wines expressed their maximum aroma potential two years after grape harvest.

## 1. Introduction

Wine aroma is produced by the interactions of hundreds of chemical compounds derived from multiple sources [1]. According to their origin, wine aroma compounds can be grape-derived such as monoterpenes and norisoprenoids [2,3]; microbially-derived secondary metabolites formed from sugar and amino acid metabolism during the fermentation [1,4]; and those compounds formed during wine storage, either in oak barrels [5,6] or in bottles [7,8]. The major groups of aroma compounds are monoterpenes, norisoprenoids, aliphatics, higher alcohols, esters, phenylpropanoids, methoxypyrazines, and volatile sulfur [2,9]. However, identifying one single compound that defines the character of a given grapevine variety has seldom been accomplished [1]. Therefore, the varietal character depends on the overall profile of odor-active compounds present in the grape and corresponding wine [1]. This character is extremely important for wine typicity and commercial success, as most wineries rely on this concept for marketing campaigns.

Wine aroma slightly evolves during bottle aging because the amounts of oxygen that penetrate through the closures are low [7,10,11]. Oxygen penetrates through the stoppers at a rate between 0.005 and 5 mg L^−1^ year^−1^ [12], depending on the type of closure used [13,14]. Small doses of oxygen may have a favorable effect on wine aroma, such as the decomposition of sulfur compounds responsible for negative flavors; however, an excess of oxygen can have adverse effects on wine aroma [14], leading to the question of how long can a given wine type be stored or aged in the bottle. In this context, the redox status can affect the release of certain varietal aromas from amino acid metabolism [15], but also can lead to the appearance of reductive aromas from sulfur compounds such as dimethyl sulfide [16].

In the northwest of the Iberian Peninsula (the regions of Galicia in Spain and Tras-dos-Montes in Portugal), white grapevine varieties are predominantly grown. Among the traditional cultivars from these regions, Treixadura is one of the most important because it is used to obtain balanced wines with a high aromatic potential [17], especially those monovarietal wines from the Ribeiro Designation of Origin (DO) in Galicia. Similar to other white wines, higher alcohols are the most important volatiles from the quantitative point of view in Treixadura wines, whereas ethyl esters, acetates, and fatty acids are qualitatively relevant for the aroma of these wines [18]. In fact, nine volatiles have a significant relevance on the aroma of Treixadura wines, including higher alcohols (2-methyl-1-butanol, 3-methyl-1-butanol, and 2-phenylethanol), acetates (isoamyl acetate and ethyl acetate), and esters (ethyl butyrate, ethyl hexanoate, ethyl octanoate, and ethyl decanoate) [18]. The concentrations of higher alcohols in Treixadura wines can be explained by the high contents in amino acids observed in grapes from this variety [19]. However, the volatile compounds that have the most relevant role in the aroma of Treixadura wines are ethyl octanoate, ethyl hexanoate, and isoamyl acetate, which provide fruity nuances [17,20]. In contrast, the contents of monoterpenes and norisoprenoids in Treixadura wines are very low and, consequently, they do not play a relevant role in the aromatic profile of the wines from this variety [17,21]. Despite being present in Treixadura wines, linalool, citronellol, and geraniol appear at low concentrations that usually do not surpass their respective odor thresholds [22]. In addition, bounded terpenes do not appear in high concentrations [23]. Finally, Treixadura wines have low concentrations of sulfur compounds, although 3-methyl-propyl acetate and 4-methyl-1-butanol may provide onion, garlic, and fungal nuances [24]. Therefore, the volatile composition of wines from this variety has been previously described under several situations [17,20,25,26]; however, no information is available about the evolution of the aroma of Treixadura wines over their storage in bottles and this leads to a debate on when the optimum time for consumption is. Furthermore, investigations monitoring the evolution of dry white wines’ flavor profiles during bottle aging are limited [7].

In this context, the aim of the current study was to assess, on a six-month basis, the alterations in the volatile composition and sensory properties of Treixadura wines from the Ribeiro DO produced over a 24-month period of bottle aging. Finally, the optimal period for consumption of Treixadura wines was determined based on the evolution of the volatile and aroma properties, providing useful information to winemakers for managing their wine stock and developing marketing campaigns.

## 2. Materials and Methods 

### 2.1. Wine Samples

Treixadura wines used in the current study, corresponding to the 2013 vintage, were made at industrial scale by several wineries from the Ribeiro DO employing their standard winemaking protocols. Bottling was performed on May 2014 at the packaging line of each winery to ensure a 750 mL volume of each bottle. Wine bottles coming from the same fermentation tank were stored in a cool place under dark conditions until analysis. All bottles had the same type of closure in order to avoid different oxygen penetration rates into the bottles. Wines were analyzed on a 6-month basis: November 2014 (M6), May 2015 (M12), November 2015 (M18), and May 2016 (M24).

### 2.2. Determination of Volatile Compounds

Methanol and higher alcohols were determined in triplicate by direct injection of 2 μL, from 5 mL of wine to which 1 mL of 4-methyl-2-pentanol (1 g L^−1^) was added as internal standard, into a Hewlett Packard 5890 gas chromatograph using an HP-Innowax capillary column (60 m × 0.25 mm i.d.; film thickness 0.25 μm) as described by Bertrand and Ribéreau-Gayon [27].

The extraction of the rest of volatile compounds was performed according to Armada et al. [28]. Briefly, a wine sample of 100 mL containing 2 mL of 3-octanol (20 mg L^−1^) and 2 mL of 3,4-dimethyl-phenol (100 mg L^−1^) as internal standards was extracted three times (10, 5, and 5 mL) with dichloromethane. Then, the organic extract was dried and concentrated to 0.5 mL under nitrogen, and 3 μL were injected in triplicate in splitless mode (purge time, 30 s; purge rate, 70) in a Hewlett Packard HP 5890-I gas chromatograph coupled to a Hewlett Packard HP 5970 mass spectrometer. Spectra were recorded in the electron impact mode (ionization energy, 70 eV; source temperature, 250 °C), using an HP-Innowax column (60 m × 0.25 mm i.d.; film thickness 0.25 μm). The carrier gas was helium (18 psi). The temperature program was isothermal at 45 °C for 1 min, then 3 °C min^−1^ to 230 °C with a final isotherm of 25 min. The acquisition was made in scanning mode (mass range, 30–300 amu; 1.9 spectra s^−1^).

The identification of the volatile compounds was confirmed by comparing their mass spectra (MS Chemstation Wiley 7N library) and their retention times with those of the pure compounds. For obtaining the calibration curves, five known amounts of the analytes were subjected to the same liquid–liquid extraction as that for the wine samples, and the quantification was carried out by the interpolation of relative peak areas with respect to the response of internal standards. Those substances for which pure compounds were not available were referred as a function of the normalized area respect to the internal standard (3-octanol). Each wine sample was analyzed in triplicate.

### 2.3. Aromatic Index

In order to estimate the influence of each volatile on the Treixadura wine aroma, odor activity values (OAV) were computed as the ratio between the concentration of a given compound and its corresponding perception threshold [29]. Theoretically, OAV should be greater than the unity [29]; however, due to synergic effects among different substances, those compounds with values greater than 0.2 can be considered as active aromas [30]. The odor thresholds for the compounds considered in this study, along with their corresponding aromatic descriptors, are shown in Table 1.

### 2.4. Sensory Evaluation

Four wine sensory assessments were carried out over the study period, each one approximately 15 days after the performance of the gas chromatography determinations. The panel consisted of 6 to 10 professional enologists (25–50 years of age, 25% females and 75% males), most of them from the wineries that supplied the wine samples. All wines were tasted in the same session, but the sessions were not replicated due to the availability of the tasting panel. The wines were served in standard tasting glasses coded with random numbers and covered with a watch-glass to minimize the loss of volatile compounds. Testing temperature was 10 °C and room temperature was 20–22 °C. A card of 7 aromatic attributes (floral, fruity, grass, spicy, woody, sulfurous, and caramel) accompanied by a scale from 0 to 10 to rate the intensity of each nuance in each wine sample, where 0 indicated that the descriptor was not perceived and 10 indicated the highest intensity. In addition, panellists must score the global quality of the wine sample both at the aroma (olfactory) and taste (mouthfeel) levels, as well as provide a global mark for the wine overall quality.

### 2.5. Statistical Analysis

Significant differences among times after bottling for the concentrations of each volatile compound were assessed with a one-way analysis of variance (ANOVA). Post-hoc comparison of means was performed using the Fischer’s Least Significant Difference (LSD) test. Similarly, ANOVA was used to determine the influence of time after bottling on the OAV of each compound. Principal Component Analysis (PCA) was applied to discriminate among the means of families of volatile compounds in the samples according to the time after bottling. Statistical analysis was carried out using R environment v.3.6.2 [41].

## 3. Results

### 3.1. Evolution of the Concentrations of Volatile Compounds Over Storage Time in the Bottle

Eight monovarietal Treixadura wines made at industrial scale were analyzed and the volatile composition of each of them is shown in the Supplementary Tables S1 to S8. A total of 44 volatiles were detected in the Treixadura wine samples studied, including terpenes, C6 compounds, higher alcohols, esters, volatile fatty acids, acetates, carbonyl compounds, volatile phenols, and other compounds, and the average value at each sampling date is displayed in Table 2. Terpenes appeared at low concentrations and the most relevant volatile within this family was linalool (Table 2). Among C6 compounds, 1-hexanol was the most quantitatively important volatile in Treixadura wines (Table 2). Isoamyl acetate and methanol were the most relevant higher alcohols detected in the samples studied (Table 2). The most relevant ester was ethyl octanoate, whereas octanoic acid was the most quantitatively important fatty acid in the Treixadura wines studied (Table 2). Finally, the most relevant volatiles among acetates, carbonyl compounds, and volatile phenols were, respectively, isoamyl acetate, acetoine, and 4-vinyl-phenol (Table 2).

Bottle storage time significantly affected the concentrations of 26 of these volatiles (Table 2). In general, concentrations were lower at the final measurement date, while no significant differences were detected among the rest of measurement dates (Table 2). Despite this lack of differences, concentrations tended to decline with storage time, except for the monoethyl and diethyl succinates and furfural, which appeared at higher concentrations on the third measurement date (Table 2).

It must be noted that not all the compounds listed in Table 2 were detected in all the Treixadura wine samples (Supplementary Tables S1 to S8). However, the main findings regarding the effect of storage time on the concentrations of volatile compounds were observed for each sample, although some exceptions to these general observations existed. In wine sample 1, the concentration of ethyl butyrate increased over time until the third date of measurements, leading to a greater content of esters on that date (Supplementary Table S1). In wine sample 2, α-terpineol was not detected and the concentrations of 2-phenylethanol and benzyl alcohol increased up to the third measurement date (Supplementary Table S2). In wine sample 3, the concentrations of volatile fatty acids were rather low but increased on the third date of measurements (Supplementary Table S3). In wine sample 4, no terpenes were detected and the concentrations of higher alcohols and acetates were lower than in the rest of the samples (Supplementary Table S4). In wine sample 5, terpenes appeared at greater concentrations on the third measurement date; the contents of higher alcohols were the greatest compared to the rest of the samples, whereas the carbonyl compounds were detected at the lowest concentrations (Supplementary Table S5). In wine sample 6, C6 compounds were detected at the lowest concentrations when compared with the rest of the samples studied; moreover, their concentrations were significantly higher on the third date of measurements (Supplementary Table S6). Wine sample 7 had the highest and lowest concentrations of terpenes and isoamyl acetate, respectively, when compared with the rest of the samples; while methanol concentration increased with storage time (Supplementary Table S7). In wine sample 8, C6 compounds appeared at low concentrations while volatile fatty acids were detected at high concentrations when compared with the rest of the samples (Supplementary Table S8).

**Figure 1:**
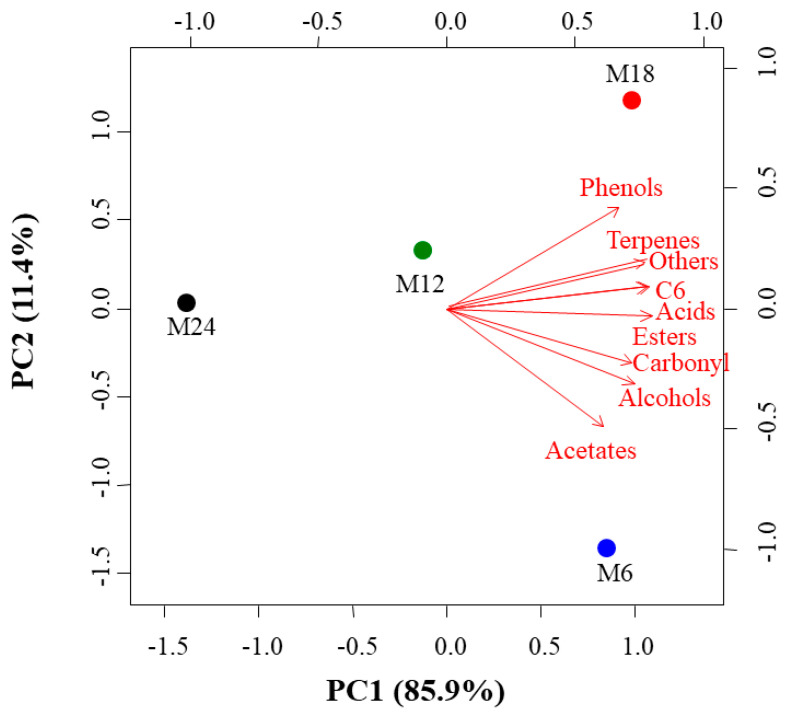
Principal component analysis (PCA) of Treixadura wines after several months of aging in the bottle: Bi-plot of the first two components (PC) for families of volatile compounds related to wine aroma. M6, M12, M18, and M24 indicate 6, 12, 18, and 24 months after bottling.

The PCA applied to the average concentrations of the different families of volatiles (Figure 1) explained 97.3% of the variability within the wine samples. The first component (PC1) explained 85.9% of this variability and depended on the concentrations of all families of compounds, whereas PC2 explained 11.4% of the variability and depended on the concentrations of phenols, terpenes, higher alcohols, and acetates (Figure 1). In the bi-plot, M6 was located on the positive side of PC1 and the negative side of PC2, due to the high concentrations of acetates in this sample. Wines from M12 were located in the center of the bi-plot, indicating that these samples did not have outstanding concentrations of any of the families of compounds. Wines from M18 were located on the positive sides of both PC due to their high concentrations of phenols and terpenes. Finally, samples from M24 were located on the negative sides of both PC, indicating that their concentrations on all the families of compounds were lower than those from the rest of the samples (Figure 1).

### 3.2. Effect of Bottle Storage Time on Odor Activity Values

Table 3 shows the OAV for the 32 volatile compounds for which odor thresholds were available. The volatiles with the highest OAV were isovaleric acid, isoamyl acetate, 4-vinyl-phenol, and ethyl hexanoate. From Table 3, a total of 21 compounds showed OAV greater than 0.2, except for the last measurement date in which 19 compounds showed OAV over this threshold. Moreover, 10 compounds had OAV greater than 1 in the first two measurement dates, 11 compounds on the third date, and 7 compounds on the last date of measurements.

The OAV of 16 compounds were significantly affected by storage time in the bottle (Table 3). In general, OAV were lower on the last date of measurements except for diethyl succinate, 4-vinyl-phenol, and 4-vinyl-guaiacol for which OAV on the last date did not significantly differ from those of the first measurement date (Table 3). In general, all wine samples showed the same profile with 20–22 volatiles with OAV greater than 0.2; from these substances, 10–12 volatiles had OAV greater than 1 (Supplementary Tables S9 and S10). Despite the fact that significant and marked reductions in the concentrations of the volatiles were detected at the end of the period of bottle storage, the reductions in OAV were less relevant. In this sense, some substances passed from having OAV greater than 1 at M6 to OAV in the range of 0.2–1 at M24; however, they can still contribute to wine aroma.

### 3.3. Evolution of the Sensory Profile of Treixadura Wines over Bottle Storage

**Figure 2:**
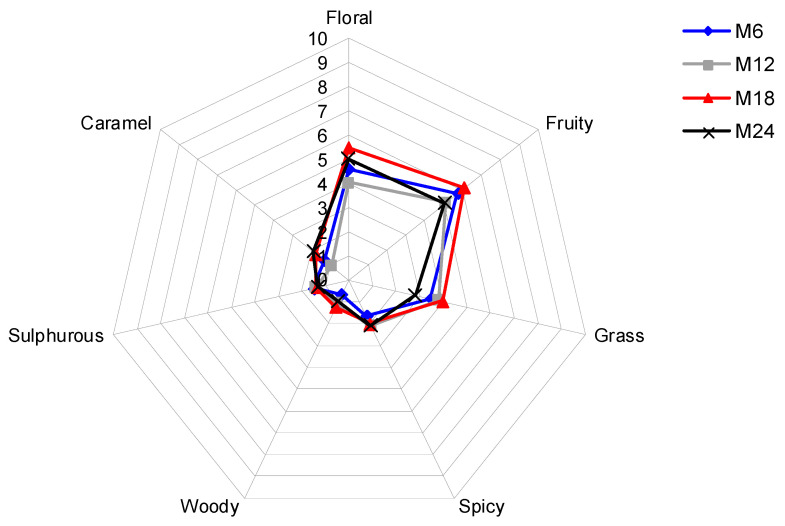
Aroma profile of Treixadura wines as affected by storage time in the bottle. Data are averages for the 8 wine samples considered in the current study. M6, M12, M18, and M24 indicate 6, 12, 18, and 24 months after bottling.

The panellists gave the highest marks to the fruity, floral and grass descriptors (Figure 2), whereas the rest of aroma descriptors did not reach more than two points in the sensory evaluations. Four descriptors (floral, fruity, grass, and caramel) showed significantly different marks depending on the storage time. In the case of floral, wines from M18 had higher marks than those from M12. In the case of fruity, wines from M18 had higher marks than those from M12 and M24. In the case of grass, wines from M24 had lower marks than those from M12 and M18. Finally, wines from M12 received lower marks for caramel than those from M18 and M24.

**Figure 3:**
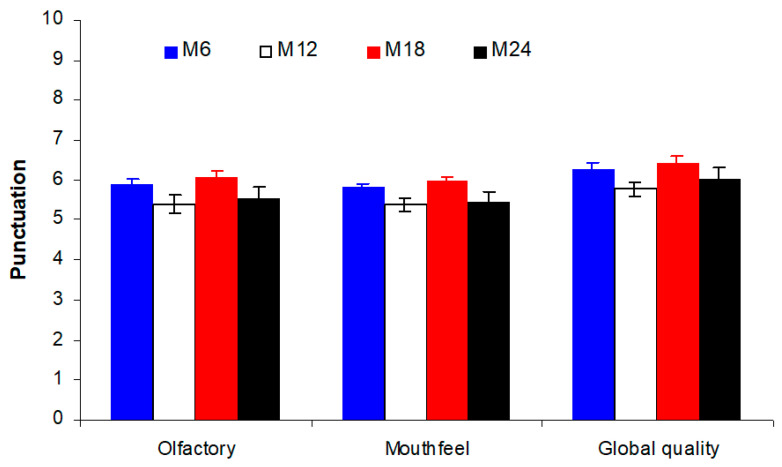
Punctuations for the olfactory and mouth phases of the sensory evaluation, as well as for the global quality of the Treixadura wines at different storage times in the bottle. Error bars represent standard errors. M6, M12, M18, and M24 indicate 6, 12, 18, and 24 months after bottling.

No significant differences among storage time in the bottle were detected for the olfactory, mouthfeel, and global marks given to the Treixadura wines, although a trend to higher marks was observed for M6 and M18 (Figure 3). Despite of a certain variability among samples, the highest global quality marks were given to samples after 18 months of bottling (M18). Some of the samples maintained these high marks six months later, but most of them suffered from a decline in this global quality mark by the end of this experiment (M24).

## 4. Discussion

This study confirmed that Treixadura wines do not have a terpenic aroma profile and, consequently, Treixadura cannot be considered rich in varietal compounds [17,20,25]. In contrast, the studied wines had high contents in ethyl esters and isoamyl acetate, which provide fruity nuances [33], and vinyl-phenols that provide aroma to paint, watercolor, and clove [40]. In the current study, Treixadura wines had a similar volatile composition at the time of bottling, despite coming from different wineries that, likely, used different protocols for winemaking. However, over the process of bottle aging, several reactions occurred and altered the volatile composition of the wines. Previous research reported that reactions such as oxidation, hydrolysis, and reactions caused by charge transfer and formation of covalent bonds influenced the evolution of wine flavor during bottle aging [42,43]. In the case of white wines, scarce research efforts have been devoted to elucidate the mechanisms that produce changes in aromatic composition during bottle aging [7]. Nevertheless, it has been suggested that an oxidation of alcohols into aldehydes is produced, as well as an increase followed by a diminishing of terpenes, acetates, and ethyl esters, while there is a formation or an increase in the concentrations of norisoprenoids, thiols, and sulfur compounds of low molecular weight [14].

In the current work, the volatile compounds detected in Treixadura wines followed one of three patterns during their evolution over bottle aging. First, the volatile compounds detected in the wines from the current study maintained their concentrations up to the third measurement date (2 years after grape harvest, 18 months in the bottle) and declined sharply in concentration on the last measurement date (30 months after grape harvest, 24 months in the bottle). Compounds relevant to wine aroma, including linalool, 2-phenylethanol, ethyl hexanoate, ethyl octanoate, isovaleric acid, and isoamyl acetate, followed this pattern over bottle aging. A second pattern was observed for the concentrations of other volatiles, which decreased steadily over the period of bottle aging, including 1,3-butanediol, isobutyric acid, isoamyl acetate, and acetoine. Finally, the concentrations of 19 volatiles did not significantly vary over the period of bottle aging (third pattern). A previous study on Cabernet Sauvignon wines pointed out similar patterns of evolution [11], although the specific pattern for a given compound differed from that observed in the current study, likely to differences in the variety and experimental setup used. In contrast, research on a white variety, Chardonnay [7], provided similar results as those presented here. In this sense, alcohols (1-hexanol, *cis*-3-hexen-1-ol, isobutanol) tended to remain stable over bottle aging, whereas ethyl esters (ethyl hexanoate, octanoate, and decanoate) and fatty acids (hexanoic, octanoic, and decanoic) tended to appear at low concentrations by the end of the bottle aging period. This diminishment of critical aroma compounds such as ethyl esters, terpenes, and norisoprenoids could reduce the perception of fruity and floral nuances at the sensory level [7,11,44]. Overall, bottle aging within 18 months enhanced the accumulation of volatile compounds and wine maturation in this study.

The changes in concentrations discussed above modified the relevance of the volatiles on wine aroma. In the current study, the compounds that had the highest OAV and, consequently, contributed significantly to Treixadura wine aroma were isovaleric acid, 4-vinyl-phenol, isoamyl acetate, and ethyl hexanoate; with OAV ranging from 2.8 to 25, depending on the compound and the date after bottling. These compounds coincide with those reported by Vilanova et al. [20] for wines of the same variety. In addition, Cortés and Blanco [18] indicated that ethyl octanoate, ethyl butyrate, isoamyl alcohol, and 2-phenylethanol also had a relevant contribution to the aroma of Treixadura wines, although their concentrations depended on the yeast strain used for fermentation. In the current study, these compounds were present and their OAV were from 0.5 (2-phenylethanol) to 4 (isoamyl alcohol), thus they contributed to wine aroma.

In this sense, the ‘fruity’ descriptor received the highest marks in all wines. In fact, several authors reported that Treixadura wines have a characteristic flavor related to fruits and pointed out several descriptors including ‘banana’, ‘apple’, ‘citrus’, and ‘pear’ [18]; ‘stone fruit’ and ‘ripen fruit’ [20]; and ‘fresh fruit’ [26]. In the current study, the ‘fruity’ descriptor received lower marks on the second date (12 months after bottling) but these marks increased six months later to decrease again on the last date of sensory evaluations. This pattern is similar to that of compounds such as ethyl esters, isoamyl acetate, and diethyl succinate, which appeared at higher concentrations on the third date of determinations and showed high OAV, which might have caused Treixadura wines to have this ‘fruity’ character. A similar behavior over bottle aging, namely, maximum values after two years from harvesting, was observed for the ‘floral’ descriptor, which might have been produced by compounds such as 2-phenylethanol and linalool. Despite the fact that these compounds appeared at OAV between 0.2 and 1, synergistic effects could have caused their detection and contributed to the Treixadura wine aroma, as previously reported [18,20,45].

The ‘grass’ descriptor received marks around 3–4 units and these were higher on the second and third dates of assessment. This nuance could be produced by isoamyl alcohols, 1-hexanol, and *cis*-3-hexenol [32,33,36], which appeared at significant concentrations in the Treixadura wines studied. These compounds are synthesized from the branched-chain amino acids [9], which are abundant in Treixadura when compared to other grape varieties [19]. The intensity of the remaining aroma descriptors was low, although some of them (‘caramel’, ‘spicy’) have been previously encountered in Treixadura wines [20,25]. Marks for ‘caramel’ increased with bottle aging, as previously reported for Riesling [46] and Semillon [47] wines, being explained by an increase of the concentration of furfural [48]; however, this was not observed in the current study. Nevertheless, the marks for this descriptor were low, up to 2 in the case of the M18 and M24 samples, and the observed increase of these marks with bottle aging can be caused by several factors such as, for instance, spontaneous malolactic fermentation or the oxidation of wine [49]; moreover, the M18 sample had a higher concentration of γ-butyrolactone, a compound that provides caramel and sweet nuances [33].

Finally, it must be noted that the nonvolatile matrix exerts a powerful impact on wine aroma perception, which has been reported similar to that of the volatile composition [50,51]. In the current study, the mouthfeel quality of the wines tended to a greater quality during the first 18 months of the bottle-aging period and this could have positively impacted the assessment of fragrance attributes, as previously reported for Cabernet Sauvignon wines [11].

## 5. Conclusions

In conclusion, by assessing the wine volatile composition, OAV, and sensory perception, a comprehensive understanding of the evolution of flavor profiles of Treixadura wines was established. Most volatile compounds in the studied wines showed stable, or even increased, concentrations up to two years after harvest (18 months of bottle aging). Then, their contents sharply decreased. The concentrations of acetates, mainly of isoamyl acetate, progressively decreased during bottle aging, being reduced up to four to five times when compared to the initial concentration in the wines. Sensory evaluation showed that the most-valued aromatic descriptors (‘fruity’ and ‘floral’) received the highest marks in the samples from 18 months of bottle aging (two years after harvest); these samples also reached the highest marks both for the olfactory and mouthfeel levels, as well as for the global quality of the wine. From the results obtained, and against the common belief that Galician white wines must be consumed within the year following their production, it would be advised that Treixadura wines were consumed two years after harvest (18 months in the bottle). Therefore, the current study has extended the research into the evolution of aroma compounds in white wines; however, further attention should be given to wine flavor chemistry and quality during bottle aging.

## Figures and Tables

**Table 1:** Odor thresholds, matrix in which they were obtained, and descriptors for several volatile compounds. References for the thresholds are included.

Family	Compound	Odor Threshold (mg L^−1^)	Matrix	Descriptor	Reference
Terpenes	linalool	0.050	Wine	Rose	[31]
	α-terpineol	0.400	Wine	Flowers, linden	
C6 Compounds	1-hexanol	4	Ethanol (11%)	Herbaceous	[32]
	*cis*-3-hexen-1-ol	1	Ethanol (10%)	Green, bitter	[33]
	*trans*-3-hexen-1-ol	13	Beer		[34]
Higher Alcohols	1-propanol	30	Not specified	Ripe fruit	[35]
	1-butanol	11	Not specified	Medicine	
	isobutanol	75	Ethanol (10%)	Clove	[33]
	isoamyl alcohol	40	Ethanol (10%)	Fusel	[36]
	2-phenylethanol	14	Ethanol (10%)	Rose, honey	[37]
Alcohols	methanol	2000	Not specified	Alcohol	[35]
	benzyl alcohol	900	Beer	Blackberry	[34]
Carbonyl Compounds	benzaldehyde	2	Ethanol (10%)	Almond	[33]
	furfural	150	Beer	Toasted	[34]
	acetoine	150	Ethanol (12%)	-	[38]
Ethyl Esters	ethyl butyrate	0.4	Ethanol (10%)	Blueberry	[33]
	ethyl hexanoate	0.08		Green apple	
	ethyl octanoate	0.58		Sweet, flower	
	ethyl decanoate	0.5		Brandy, grape	
	ethyl lactate	150		Butter	
	diethyl succinate	1.2		Melon	
Acetates of Higher Alcohols	isoamyl acetate	0.16	Ethanol (10%)	Banana	[33]
	hexyl acetate	0.67		Pear, apple, cherry	
	2-phenylethyl acetate	1.8		Rose, flower	
Volatile Fatty Acids	butyric acid	4	Ethanol (9.5%)	Butter, cheese	[39]
	isobutyric acid	2.3	Ethanol (11%)	-	[37]
	isovaleric acid	0.03		-	
	hexanoic acid	3	Beer	Cheese, fatty	[34]
	octanoic acid	10		Fatty, rancid	
	decanoic acid	6			
Volatile Phenols	4-vinyl-guaiacol	0.440	Ethanol (12%)	Paint, watercolor	[40]
	4-vinyl-phenol	0.375		Pharmacy, clove	

**Table 2:** Average concentrations of volatile compounds (mean ± standard error) in Treixadura wines from the Ribeiro Designation of Origin (DO) at different times of bottle aging. M6, M12, M18, and M24 indicate 6, 12, 18, and 24 months after bottling.

Family	Compound ^1^	M6	M12	M18	M24	*P*-Value ^1^
Terpenes	linalool *	29.6 ± 2.4 a	29.7 ± 1.0 a	32.6 ± 1.6 a	18.4 ± 0.7 b	0.003
	α-terpineol *	21.9 ± 5.2	22.0 ± 6.8	34.9 ± 5.4	16.7 ± 2.2	0.597
C6 Compounds	1-hexanol	1.3 ± 0.1 a	1.1 ± 0.1 ab	1.4 ± 0.1 a	0.7 ± 0.0 b	0.011
	*cis*-3-hexen-1-ol	0.22 ± 0.05	0.18 ± 0.04	0.19 ± 0.04	0.11 ± 0.02	0.736
	*trans*-3-hexen-1-ol	0.20 ± 0.08	0.26 ± 0.11	0.28 ± 0.12	0.11 ± 0.04	0.861
Alcohols	methanol	63.3 ± 3.5	58.4 ± 2.3	63.4 ± 2.6	63.1 ± 2.9	0.710
	1-propanol	15.1 ± 1.3	12.8 ± 1.0	13.3 ± 1.4	16.1 ± 1.4	0.494
	isobutanol	20.2 ± 1.2	19.2 ± 1.1	22.3 ± 1.6	17.9 ± 1.2	0.297
	1-butanol	1.3 ± 0.1	1.3 ± 0.1	1.3 ± 0.1	0.9 ± 0.1	0.134
	isoamyl alcohol	164.3 ± 6.9	158.0 ± 7.2	159.5 ± 8.0	152.3 ± 6.4	0.824
	benzyl alcohol	0.08 ± 0.01	0.29 ± 0.08	0.21 ± 0.02	0.09 ± 0.01	0.129
	2-phenylethanol	8.5 ± 0.5 a	7.8 ± 0.3 a	9.1 ± 0.5 a	3.7 ± 0.3 a	<0.001
	3-methyl-1-pentanol	0.07 ± 0.01 ab	0.07 ± 0.00 ab	0.07 ± 0.00 a	0.04 ± 0.00 b	0.031
	3-ethoxy-1-propanol #	38.3 ± 7.9	38.9 ± 7.0	35.1 ± 7.2	19.9 ± 3.9	0.456
	1,2-propanodiol #	8.6 ± 0.7	8.0 ± 0.3	7.4 ± 0.7	4.8 ± 0.2	0.221
	1,3-butanediol #	355.8 ± 35.1 a	258.9 ± 15.1 ab	247.1 ± 19.8 ab	132.8 ± 15.8 b	<0.001
	2,3-butanediol #	78.9 ± 7.2 a	66.0 ± 5.9 a	59.5 ± 4.1 ab	32.9 ± 3.7 b	0.003
Ethyl Esters	ethyl butyrate	0.44 ± 0.04	0.39 ± 0.03	0.44 ± 0.03	0.31 ± 0.02	0.167
	ethyl hexanoate	0.65 ± 0.03 a	0.54 ± 0.05 a	0.63 ± 0.02 a	0.35 ± 0.02 b	<0.001
	ethyl octanoate	1.41 ± 0.13 a	1.48 ± 0.06 a	1.21 ± 0.05 a	0.63 ± 0.02 b	<0.001
	ethyl decanoate	0.58 ± 0.02 a	0.63 ± 0.04 a	0.63 ± 0.02 a	0.30 ± 0.01 b	<0.001
	ethyl-3-hydroxybutyrate	0.14 ± 0.01 a	0.14 ± 0.01 a	0.16 ± 0.01 a	0.08 ± 0.01 b	0.006
	ethyl-4-hydroxybutyrate #	92.7 ± 13.6 a	62.7 ± 6.3 ab	56.4 ± 6.6 ab	22.3 ± 2.2 b	0.003
	ethyl lactate	13.8 ± 1.7	9.7 ± 0.5	15.1 ± 2.1	8.4 ± 1.3	0.170
	monoethyl succinate #	50.3 ± 3.0 b	54.2 ± 2.5 b	79.3 ± 3.9 a	27.7 ± 1.4 c	<0.001
	diethyl succinate	0.78 ± 0.06 b	1.16 ± 0.11 b	1.80 ± 0.11 a	0.85 ± 0.08 b	<0.001
Volatile Fatty Acids	isobutyric acid	1.01 ± 0.08 a	0.93 ± 0.07 a	0.93 ± 0.06 a	0.56 ± 0.03 b	0.002
	butyric acid	2.88 ± 0.13 a	2.75 ± 0.11 a	2.90 ± 0.13 a	1.62 ± 0.14 b	<0.001
	isovaleric acid	0.75 ± 0.04 a	0.73 ± 0.02 a	0.74 ± 0.03 a	0.40 ± 0.02 b	<0.001
	hexanoic acid	4.7 ± 0.1 a	4.2 ± 0.1 a	4.7 ± 0.1 a	2.2 ± 0.1 b	<0.001
	octanoic acid	6.4 ± 0.2 a	6.1 ± 0.2 a	6.9 ± 0.1 a	3.2 ± 0.1 b	<0.001
	decanoic acid	2.1 ± 0.1 a	2.0 ± 0.1 a	2.1 ± 0.0 a	0.9 ± 0.0 b	<0.001
	lauric acid	0.16 ± 0.02	0.16 ± 0.02	0.15 ± 0.02	0.18 ± 0.07	0.987
	*trans*-2-hexenoic acid #	16.6 ± 1.9	16.7 ± 2.2	19.0 ± 1.9	11.2 ± 0.9	0.262
Acetates of Higher Alcohols	isoamyl acetate	2.1 ± 0.2 a	1.4 ± 0.1 a	1.0 ± 0.1 a	0.5 ± 0.1 b	<0.001
	hexyl acetate	0.14 ± 0.02	0.17 ± 0.05	0.15 ± 0.04	0.08 ± 0.02	0.738
	2-phenylethyl acetate	0.10 ± 0.01 a	0.09 ± 0.01 a	0.07 ± 0.01 ab	0.02 ± 0.00 b	0.003
Carbonyl Compounds	furfural	0.03 ± 0.00 c	0.04 ± 0.00 bc	0.07 ± 0.01 a	0.05 ± 0.00 b	<0.001
	benzaldehyde	0.02 ± 0.00	0.03 ± 0.00	0.03 ± 0.0	0.01 ± 0.00	0.126
	acetoine	3.1 ± 0.5	2.1 ± 0.2	2.6 ± 0.4	1.8 ± 0.3	0.427
Volatile Phenols	4-vinyl-phenol	3.8 ± 0.3 b	5.5 ± 0.4 ab	5.7 ± 0.4 a	1.9 ± 0.1 c	<0.001
	4-vinyl-guaiacol	1.4 ± 0.1 b	1.6 ± 0.1 ab	2.0 ± 0.1 a	0.5 ± 0.0 c	<0.001
Others	γ-butyrolactone	1.8 ± 0.2 ab	1.4 ± 0.1 ab	1.9 ± 0.2 a	1.0 ± 0.1 b	0.016
	methionol #	34.2 ± 2.4 a	34.1 ± 2.0 a	37.0 ± 2.1 a	16.4 ± 1.4 b	<0.001

^1^ Concentrations in mg L^−1^; except for those compounds marked with * in μg L^−1^ and # as normalized area. Different letters in the row indicate significant differences among times after bottling for a given compound.

**Table 3:** Odor activity values of volatile compounds in Treixadura wines from the Ribeiro DO at different times of bottle aging. M6, M12, M18, and M24 indicate 6, 12, 18, and 24 months after bottling.

Family	Compound	M6	M12	M18	M24	*p*-Value
Terpenes	linalool ^1^	0.6 a	0.6 a	0.7 a	0.4 b	0.006
	α-terpineol	0.0	0.0	0.1	0.0	0.301
C6 Compounds	1-hexanol	0.3 ab	0.3 ab	0.4 a	0.2 b	0.008
	*cis*-3-hexen-1-ol	0.2	0.2	0.2	0.2	0.994
	*trans*-3-hexen-1-ol	0.0	0.0	0.0	0.0	0.819
Alcohols	methanol	0.0	0.0	0.0	0.0	0.999
	1-propanol	0.5	0.4	0.5	0.5	0.572
	isobutanol	0.3	0.3	0.3	0.2	0.332
	1-butanol	0.1	0.1	0.1	0.1	0.240
	isoamyl alcohol	4.1	4.0	4.0	3.8	0.781
	benzyl alcohol	0.0	0.0	0.0	0.0	0.999
	2-phenylethanol	0.6 a	0.5 a	0.6 a	0.3 b	<0.001
Ethyl Esters	ethyl butyrate	1.1	1.0	1.1	0.8	0.306
	ethyl hexanoate	8.2 a	6.7 ab	7.8 a	4.8 b	0.003
	ethyl octanoate	2.5 a	2.5 a	2.1 a	1.2 b	<0.001
	ethyl decanoate	1.2 a	1.3 a	1.3 a	0.7 b	0.001
	ethyl lactate	0.1 a	0.1 a	0.1 a	0.0 b	0.003
	diethyl succinate	0.7 b	1.0 a	1.5 a	0.7 b	<0.001
Volatile Fatty Acids	isobutyric acid	0.5 a	0.4 a	0.4 a	0.2 b	0.002
	butyric acid	0.7 a	0.7 a	0.7 a	0.4 b	<0.001
	isovaleric acid	25.1 a	24.4 a	24.7 a	13.3 b	<0.001
	hexanoic acid	1.6 a	1.4 a	1.6 a	0.7 b	<0.001
	octanoic acid	0.6 a	0.6 a	0.7 a	0.3 b	<0.001
	decanoic acid	0.4	1.1	1.1	0.1	0.471
Acetates of Higher Alcohols	isoamyl acetate	13.3 a	8.7 ab	6.1 bc	2.8 c	<0.001
	hexyl acetate	0.2	0.2	0.2	0.1	0.877
	2-phenylethyl acetate	0.1	0.1	0.0	0.0	0.059
Carbonyl Compounds	furfural	0.0	0.0	0.0	0.0	0.999
	benzaldehyde	0.0	0.0	0.0	0.0	0.999
	acetoine	0.0	0.0	0.0	0.0	0.451
Volatile Phenols	4-vinyl-phenol	10.2 b	14.7 ab	15.2 a	5.2 c	<0.001
	4-vinyl-guaiacol	3.2 b	3.6 b	4.6 a	1.1 c	<0.001

^1^ Different letters in the row indicate significant differences among times after bottling for a given compound.

## Supplementary Materials

The following are available online at https://www.mdpi.com/2304-8158/9/10/1419/s1, Tables S1 to S8: Concentrations of volatile compounds (mean ± standard deviation SD) in Treixadura wine samples 1 to 8 from the Ribeiro DO at different times of bottle aging, Table S9: Bottling storage effects on the odor activity values of wines from Treixadura in Ribeiro Designation of Origin (Samples 1–4), Table S10: Bottling storage effects on the odor activity values of wines from Treixadura in Ribeiro Designation of Origin (samples 5–8).
