# Supplementary material for: Evolution of the Aroma of Treixadura Wines during Bottle Aging
Source: Foods. 2020 Oct 8;9(10):1419. doi: 10.3390/foods9101419 (PMC7600726; doi:10.3390/foods9101419)
Supplement: Supplementary file 1 [file foods-09-01419-s001.pdf]

SUPPLEMENTARY MATERIAL

Table S1. Concentrations of volatile compounds (mean ± standard deviation SD) in Treixadura wine sample 1 from the Ribeiro DO at different times of bottle aging. M6, M12, M18 and M24 indicate 6, 12, 18 and 24 months after bottling.

WINE 1		M6		M12		M18		M24	
		Mean	SD	Mean	SD	Mean	SD	Mean	SD
Terpenes	linalool *	27.5 ^{b}	2.5	30.4 ^{b}	0.0	26.7 ^{b}	1.1	16.1 ^{a}	1.6
	α-terpineol *	11.3 ^{a}	0.5	10.7 ^{a}	0.0	25.4 ^{b}	2.1	13.1 ^{a}	0.2
C6 Compounds	1-hexanol	1.25 ^{b}	0.03	1.14 ^{b}	0.01	1.21 ^{b}	0.00	0.689 ^{a}	0.008
	cis-3-hexen-1-ol	0.218 ^{c}	0.013	0.128 ^{b}	0.002	0.142 ^{b}	0.001	0.083 ^{a}	0.001
	trans-3-hexen-1-ol	0.078 ^{b}	0.004	0.088 ^{c}	0.006	0.096 ^{d}	0.001	0.055 ^{a}	0.004
Alcohols	methanol	44.5 ^{a}	1.4	48.1 ^{a}	0.2	73.1 ^{c}	1.9	55.2 ^{b}	2.6
	1-propanol	14.3 ^{bc}	0.3	13.1 ^{ab}	0.7	10.7 ^{a}	0.6	15.2 ^{c}	2.1
	isobutanol	17.6 ^{a}	0.1	17.6 ^{a}	0.3	26.9 ^{b}	0.9	17.7 ^{a}	0.5
	1-butanol	1.61 ^{ab}	0.17	1.74 ^{b}	0.01	1.53 ^{ab}	0.16	1.30 ^{a}	0.06
	isoamyl alcohol	160 ^{c}	2	155 ^{b}	2	131 ^{a}	2	159 ^{bc}	2
	benzyl alcohol	0.075 ^{a}	0.008	0.124 ^{b}	0.001	0.166 ^{c}	0.012	0.077 ^{a}	0.006
	2-phenylethanol	8.32 ^{c}	0.26	7.69 ^{b}	0.02	8.64 ^{c}	0.01	3.50 ^{a}	0.02
	3-methyl-1-pentanol	0.063 ^{b}	0.005	0.072 ^{c}	0.001	0.082 ^{d}	0.001	0.046 ^{a}	0.001
	3-ethoxy-1-propanol#	94.3 ^{d}	2.1	90.1 ^{c}	0.3	85.4 ^{b}	0.3	55.2 ^{a}	0.6
	1,2-propanodiol#	8.21 ^{b}	0.21	7.54 ^{b}	0.68	6.57 ^{ab}	1.27	4.70 ^{a}	0.10
	1,3-butanediol#	433 ^{d}	14	272 ^{b}	5	321 ^{c}	1	178 ^{a}	1
	2,3-butanediol#	93.9 ^{c}	1.9	75.0 ^{b}	2.0	72.7 ^{b}	1.4	40.9 ^{a}	0.1
Esters	ethyl butyrate	0.063 ^{a}	0.042	0.203 ^{b}	0.009	0.623 ^{d}	0.015	0.426 ^{c}	0.022
	ethyl hexanoate	0.803 ^{c}	0.019	0.742 ^{b}	0.004	0.738 ^{b}	0.004	0.422 ^{a}	0.002
	ethyl octanoate	1.26 ^{b}	0.04	1.82 ^{d}	0.01	1.36 ^{c}	0.03	0.752 ^{a}	0.007
	ethyl decanoate	0.597 ^{c}	0.023	0.499 ^{b}	0.002	0.687 ^{d}	0.008	0.328 ^{a}	0.004
	ethyl-3-hydroxybutyrate	0.134 ^{b}	0.009	0.139 ^{b}	0.003	0.140 ^{b}	0.005	0.083 ^{a}	0.002
	ethyl-4-hydroxybutyrate#	78.0 ^{d}	3.0	60.0 ^{c}	1.0	45.9 ^{b}	1.0	20.8 ^{a}	0.0
	ethyl lactate	9.89 ^{c}	0.31	8.74 ^{b}	0.05	10.30 ^{c}	0.04	6.23 ^{a}	0.02
	monoethyl succinate#	39.2 ^{b}	1.3	48.2 ^{c}	1.0	63.6 ^{d}	0.3	25.4 ^{a}	0.1
	diethyl succinate	0.600 ^{b}	0.020	0.838 ^{c}	0.004	1.49 ^{d}	0.00	0.328 ^{a}	0.003
Volatile Fatty Acids	isobutyric acid	0.687 ^{b}	0.048	0.679 ^{b}	0.013	0.676 ^{b}	0.001	0.432 ^{a}	0.003
	butyric acid	3.13 ^{b}	0.13	3.18 ^{bc}	0.01	3.35 ^{c}	0.03	2.94 ^{a}	0.00
	isovaleric acid	0.690 ^{c}	0.005	0.673 ^{b}	0.002	0.715 ^{d}	0.015	0.412 ^{a}	0.003
	hexanoic acid	5.48 ^{c}	0.19	5.06 ^{b}	0.03	5.30 ^{bc}	0.02	2.48 ^{a}	0.01
	octanoic acid	7.21 ^{b}	0.23	6.98 ^{b}	0.04	7.60 ^{c}	0.03	3.36 ^{a}	0.01
	decanoic acid	2.11 ^{bc}	0.04	2.06 ^{b}	0.01	2.13 ^{c}	0.01	0.844 ^{a}	0.003
	lauric acid	0.193 ^{c}	0.004	0.128 ^{b}	0.000	0.136 ^{b}	0.011	0.042 ^{a}	0.005
	trans-2-hexenoic acid#	10.2 ^{b}	0.6	10.8 ^{b}	0.1	15.7 ^{c}	0.4	8.1 ^{a}	0.3
Acetates of Higher Alcohols	isoamyl acetate	2.15 ^{d}	0.06	1.52 ^{c}	0.01	0.910 ^{b}	0.000	0.422 ^{a}	0.004
	hexyl acetate	0.148 ^{c}	0.004	0.094 ^{b}	0.040	0.072 ^{ab}	0.001	0.043 ^{a}	0.002
	2-phenylethyl acetate	0.099 ^{d}	0.003	0.083 ^{c}	0.002	0.064 ^{b}	0.001	0.023 ^{a}	0.001
Carbonyl Compounds	furfural	0.029 ^{a}	0.001	0.041 ^{b}	0.005	0.081 ^{d}	0.002	0.055 ^{c}	0.002
	benzaldehyde	0.026 ^{b}	0.001	0.029 ^{b}	0.002	0.029 ^{b}	0.002	0.013 ^{a}	0.001
	acetoine	3.29 ^{c}	0.10	3.31 ^{c}	0.13	2.97 ^{b}	0.02	2.12 ^{a}	0.13
Volatile Phenols	4-vinyl-phenol	2.50 ^{a}	0.10	3.29 ^{b}	0.08	5.75 ^{c}	0.39	1.96 ^{a}	0.08
	4-vinyl-guaiacol	1.18 ^{b}	0.27	1.70 ^{c}	0.07	2.09 ^{d}	0.01	0.50 ^{a}	0.03
Others	γ-butyrolactone	1.71 ^{c}	0.09	1.52 ^{b}	0.01	1.68 ^{c}	0.00	0.80 ^{a}	0.00
	methionol#	23.7 ^{b}	1.1	23.0 ^{b}	0.3	30.0 ^{c}	0.8	13.0 ^{a}	0.5

Concentrations en mg L^{-1}; * en µg L^{-1}; # as normalized area.
SD: Standard deviation of three replicates.
Different letters in the row indicate significant differences among sampling dates for a given compound at $p < 0.05$.

Table S2. Concentrations of volatile compounds (mean ± standard deviation SD) in Treixadura wine sample 2 from the Ribeiro DO at different times of bottle aging. M6, M12, M18 and M24 indicate 6, 12, 18 and 24 months after bottling.

WINE 2		M6		M12		M18		M24	
		Mean	SD	Mean	SD	Mean	SD	Mean	SD
Terpenes	linalool *	27.5 [a]	1.0	31.9 [b]	1.0	30.9 [b]	2.6	-	-
C6 Compounds	1-hexanol	1.58 [c]	0.03	1.39 [a]	0.00	1.50 [b]	0.01	-	-
	cis-3-hexen-1-ol	0.095 [a]	0.009	0.106 [a]	0.001	0.117 [a]	0.000	-	-
	trans-3-hexen-1-ol	0.097 [a]	0.013	0.091 [a]	0.000	0.103 [a]	0.005	-	-
Alcohols	methanol	78.8 [c]	2.9	65.6 [b]	3.9	55.1 [a]	1.1	65.3 [b]	2.3
	1-propanol	21.1 [b]	1.6	17.1 [ab]	1.6	14.8 [a]	2.6	20.3 [b]	1.2
	isobutanol	19.1 [b]	0.9	15.8 [a]	1.6	17.3 [a]	0.1	15.1 [a]	1.1
	1-butanol	1.27 [a]	0.09	1.14 [a]	0.08	1.30 [a]	0.10	-	-
	isoamyl alcohol	190 [c]	6	176 [b]	6	162 [a]	1	172 [ab]	2
	benzyl alcohol	0.112 [a]	0.008	0.245 [b]	0.000	0.366 [c]	0.009	-	-
	2-phenylethanol	9.69 [b]	0.08	8.27 [a]	0.01	10.80 [c]	0.10	-	-
	3-methyl-1-pentanol	0.080 [a]	0.010	0.074 [a]	0.002	0.074 [a]	0.003	-	-
	3-ethoxy-1-propanol#	60.8 [b]	3.7	52.0 [a]	0.1	52.9 [a]	0.4	-	-
	1,2-propanodiol#	8.87 [c]	0.54	7.35 [b]	0.34	5.64 [a]	0.53	-	-
	1,3-butanediol#	391 [c]	5	290 [b]	1	250 [a]	2	-	-
	2,3-butanediol#	82.2 [c]	1.2	64.9 [b]	0.2	52.7 [a]	0.8	-	-
Esters	ethyl butyrate	0.638 [c]	0.044	0.484 [b]	0.020	0.248 [a]	0.000	-	-
	ethyl hexanoate	0.480 [b]	0.016	0.431 [a]	0.035	0.659 [c]	0.009	-	-
	ethyl octanoate	1.85 [c]	0.01	1.62 [b]	0.00	0.965 [a]	0.010	-	-
	ethyl decanoate	0.529 [a]	0.020	0.663 [c]	0.004	0.597 [b]	0.002	-	-
	ethyl-3-hydroxybutyrate	0.178 [a]	0.010	0.179 [a]	0.005	0.186 [a]	0.007	-	-
	ethyl-4-hydroxybutyrate#	71.4 [c]	2.1	57.1 [b]	1.7	45.9 [a]	1.1	-	-
	ethyl lactate	19.2 [c]	0.1	12.8 [a]	0.0	15.9 [b]	0.1	-	-
	monoethyl succinate#	62.2 [a]	1.7	62.7 [a]	0.6	95.3 [b]	0.6	-	-
	diethyl succinate	1.02 [a]	0.01	1.29 [b]	0.00	2.28 [c]	0.02	-	-
Volatile Fatty Acids	isobutyric acid	0.734 [a]	0.051	0.646 [a]	0.011	0.651 [a]	0.032	-	-
	butyric acid	3.84 [c]	0.08	3.38 [b]	0.01	3.70 [a]	0.05	-	-
	isovaleric acid	0.742 [c]	0.009	0.647 [b]	0.003	0.687 [a]	0.019	-	-
	hexanoic acid	5.02 [b]	0.05	4.23 [a]	0.01	4.97 [b]	0.04	-	-
	octanoic acid	5.30 [a]	0.10	5.96 [b]	0.06	7.03 [c]	0.06	-	-
	decanoic acid	1.76 [a]	0.02	1.98 [b]	0.01	1.99 [b]	0.02	-	-
	lauric acid	0.143 [b]	0.004	0.125 [a]	0.011	0.126 [a]	0.005	-	-
	trans-2-hexenoic acid#	11.3 [a]	0.8	11.6 [a]	0.3	11.9 [a]	0.2	-	-
Acetates of Higher Alcohols	isoamyl acetate	2.55 [c]	0.03	1.56 [b]	0.00	1.09 [a]	0.01	-	-
	hexyl acetate	0.182 [b]	0.004	0.093 [a]	0.051	0.087 [a]	0.002	-	-
	2-phenylethyl acetate	0.106 [b]	0.007	0.096 [b]	0.002	0.070 [a]	0.001	-	-
Carbonyl Compounds	furfural	0.026 [a]	0.001	0.032 [a]	0.001	0.048 [b]	0.004	-	-
	benzaldehyde	0.016 [a]	0.00	0.017 [a]	0.001	0.017 [a]	0.002	-	-
	acetoine	1.05 [c]	0.00	0.808 [a]	0.023	0.936 [b]	0.020	-	-
Volatile Phenols	4-vinyl-phenol	3.65 [a]	0.10	7.56 [c]	0.61	6.02 [b]	0.29	-	-
	4-vinyl-guaiacol	1.71 [a]	0.27	1.77 [a]	0.02	1.68 [a]	1.25	-	-
Others	γ-butyrolactone	1.91 [b]	0.02	1.62 [a]	0.00	1.90 [b]	0.02	-	-
	methionol#	36.0 [a]	2.0	35.2 [a]	1	37.8 [a]	0.5	-	-

Concentrations en mg L^{-1}; * en µg L^{-1}; # as normalized area.
SD: Standard deviation of three replicates.
Different letters in the row indicate significant differences among sampling dates for a given compound at $p < 0.05$.

Table S3. Concentrations of volatile compounds (mean ± standard deviation SD) in Treixadura wine sample 3 from the Ribeiro DO at different times of bottle aging. M6, M12, M18 and M24 indicate 6, 12, 18 and 24 months after bottling.

WINE 3		M6		M12		M18		M24	
		Mean	SD	Mean	SD	Mean	SD	Mean	SD
Terpenes	linalool *	25.9 b	0.5	29.8 c	1.0	24.1 b	0.2	19.0 a	1.7
C6 Compounds	1-hexanol	1.21 c	0.00	1.13 b	0.00	1.16 b	0.03	0.703 a	0.022
	cis-3-hexen-1-ol	0.781 d	0.001	0.572 b	0.001	0.621 c	0.001	0.346 a	0.001
	trans-3-hexen-1-ol	1.08 b	0.00	1.32 c	0.00	1.69 d	0.01	0.463 a	0.006
Alcohols	methanol	80.8 c	1.1	52.4 a	0.2	65.3 b	2.9	78.5 c	3.2
	1-propanol	20.9 b	2.1	12.7 a	2.1	14.2 a	1.9	15.7 a	1.3
	isobutanol	22.5 b	1.2	20.0 ab	0.4	20.9 ab	0.2	20.8 a	0.4
	1-butanol	1.94 b	0.21	1.80 ab	0.14	1.61 ab	0.20	1.27 a	0.27
	isoamyl alcohol	163 b	8	143 a	2	154 ab	2	152 ab	4
	benzyl alcohol	0.055 a	0.001	0.101 b	0.003	0.244 c	0.019	0.078 a	0.008
	2-phenylethanol	7.75 c	0.04	6.86 b	0.06	8.24 c	0.42	3.48 a	0.10
	3-methyl-1-pentanol	0.042 b	0.003	0.044 b	0.001	0.044 b	0.006	0.028 a	0.003
	3-ethoxy-1-propanol#	25.1 b	1.0	24.7 b	0.6	22.1 b	2.0	14.8 a	0.6
	1,2-propanodiol#	12.4 c	1.2	9.17 b	0.87	11.10 bc	0.54	5.82 a	0.60
	1,3-butanediol#	640 d	1	327 b	3	358 c	10	244 a	6
	2,3-butanediol#	142.0 d	2.0	115.0 c	1.0	85.6 b	2.0	61.0 a	2.0
Esters	ethyl butyrate	0.524 b	0.043	0.420 ab	0.008	0.371 a	0.074	0.338 a	0.019
	ethyl hexanoate	0.553 c	0.022	0.490 bc	0.001	0.464 b	0.058	0.267 a	0.007
	ethyl octanoate	1.89 d	0.01	1.26 c	0.01	1.09 b	0.04	0.544 a	0.020
	ethyl decanoate	0.430 b	0.020	0.458 b	0.006	0.532 c	0.023	0.269 a	0.010
	ethyl-3-hydroxybutyrate	0.180 b	0.002	0.185 b	0.000	0.199 b	0.011	0.104 a	0.005
	ethyl-4-hydroxybutyrate#	51.2 d	1.0	42.0 c	2.0	29.6 b	1.1	13.1 a	1.5
	ethyl lactate	11.4 c	0.1	10.0 b	0.1	12.1 c	0.5	6.8 a	0.2
	monoethyl succinate#	48.7 b	0.3	50.3 c	0.4	77.9 d	0.5	29.9 a	0.6
	diethyl succinate	0.645 a	0.003	0.938 b	0.006	1.720 c	0.087	0.982 d	0.031
Volatile Fatty Acids	isobutyric acid	1.04 b	0.02	1.00 b	0.01	0.977 b	0.038	0.565 a	0.011
	butyric acid	2.30 bc	0.00	2.27 b	0.01	2.43 c	0.10	1.23 a	0.02
	isovaleric acid	0.709 b	0.003	0.672 b	0.003	0.672 b	0.039	0.377 a	0.006
	hexanoic acid	3.67 c	0.03	3.23 b	0.03	3.72 c	0.18	1.85 a	0.05
	octanoic acid	5.14 c	0.02	4.71 b	0.03	5.75 d	0.28	2.75 a	0.08
	decanoic acid	1.66 bc	0.03	1.54 b	0.01	1.77 c	0.09	0.860 a	0.020
	lauric acid	0.231 b	0.016	0.149 a	0.022	0.140 a	0.003	0.600 c	0.003
	trans-2-hexenoic acid#	13.0 b	0.0	14.9 c	0.5	15.0 c	1.0	10.3 a	0.3
Acetates of Higher Alcohols	isoamyl acetate	2.90 d	0.00	2.07 c	0.01	1.34 b	0.06	0.674 a	0.020
	hexyl acetate	0.203 c	0.004	0.084 b	0.020	0.104 b	0.009	0.057 a	0.002
	2-phenylethyl acetate	0.143 d	0.002	0.131 c	0.003	0.103 b	0.006	0.04 a	0.002
Carbonyl Compounds	furfural	0.025 a	0.019	0.046 a	0.001	0.075 a	0.006	0.047 a	0.002
	benzaldehyde	0.019 b	0.001	0.019 b	0.001	0.019 b	0.001	0.008 a	0.000
	acetoine	2.30 c	0.10	2.15 c	0.11	1.90 b	0.00	1.24 a	0.05
Volatile Phenols	4-vinyl-phenol	4.54 b	0.79	5.94 c	0.33	4.78 bc	0.23	1.56 a	0.09
	4-vinyl-guaiacol	1.08 b	0.12	1.32 c	0.05	1.70 d	0.11	0.463 a	0.012
Others	γ-butyrolactone	1.06 c	0.01	0.970 b	0.000	1.16 d	0.06	0.507 a	0.018
	methionol#	38.5 b	2.3	35.1 b	1.0	38.1 b	1.7	19.4 a	0.9

Concentrations en mg L^{-1}; * en µg L^{-1}; # as normalized area.
SD: Standard deviation of three replicates.
Different letters in the row indicate significant differences among sampling dates for a given compound at $p < 0.05$.

Table S4. Concentrations of volatile compounds (mean ± standard deviation SD) in Treixadura wine sample 4 from the Ribeiro DO at different times of bottle aging. M6, M12, M18 and M24 indicate 6, 12, 18 and 24 months after bottling.

WINE 4		M6		M12		M18		M24	
		Mean	SD	Mean	SD	Mean	SD	Mean	SD
C6 Compounds	1-hexanol	1.26 [c]	0.01	1.16 [b]	0.00	1.14 [b]	0.01	0.690 [a]	0.010
	cis-3-hexen-1-ol	0.139 [b]	0.001	0.133 [b]	0.001	0.155 [c]	0.003	0.091 [a]	0.003
	trans-3-hexen-1-ol	0.073 [b]	0.004	0.081 [b]	0.005	0.076 [b]	0.002	0.054 [a]	0.003
Alcohols	methanol	47.5 [a]	0.7	49.9 [ab]	1.9	53.4 [b]	2.3	64.1 [c]	2.4
	1-propanol	8.9 [a]	0.9	8.7 [a]	2.0	9.3 [a]	0.4	11.2 [a]	1.4
	isobutanol	14.3 [a]	0.6	14.7 [a]	1.4	27.1 [c]	1.9	22.7 [b]	0.9
	1-butanol	1.02 [a]	0.17	0.799 [a]	0.022	1.01 [a]	0.07	0.701 [a]	0.168
	isoamyl alcohol	128 [a]	5	127 [a]	3	133 [a]	1	185 [b]	4
	benzyl alcohol	0.049 [a]	0.004	0.089 [b]	0.002	0.157 [c]	0.004	0.053 [a]	0.006
	2-phenylethanol	5.69 [c]	0.05	5.48 [b]	0.02	6.27 [d]	0.10	2.40 [a]	0.01
	3-methyl-1-pentanol	0.060 [b]	0.002	0.058 [ab]	0.003	0.057 [ab]	0.003	0.044 [a]	0.003
	3-ethoxy-1-propanol#	14.8 [b]	0.1	14.4 [b]	0.6	14.1 [b]	1.1	8.88 [a]	0.89
	1,3-butanediol#	206 [d]	0	157 [c]	1	152 [b]	1	80.6 [a]	1.4
	2,3-butanediol#	48.1 [c]	2.3	37.2 [b]	0.4	39.9 [b]	0.6	22.2 [a]	0.2
Esters	ethyl butyrate	0.383 [b]	0.017	0.234 [a]	0.012	0.390 [b]	0.035	0.276 [a]	0.013
	ethyl hexanoate	0.701 [d]	0.011	0.635 [c]	0.004	0.612 [b]	0.007	0.370 [a]	0.006
	ethyl octanoate	0.925 [b]	0.009	1.41 [d]	0.01	1.04 [c]	0.01	0.626 [a]	0.009
	ethyl decanoate	0.597 [b]	0.005	0.581 [b]	0.001	0.618 [b]	0.024	0.320 [a]	0.004
	ethyl-3-hydroxybutyrate	0.051 [b]	0.001	0.058 [c]	0.002	0.062 [c]	0.002	0.033 [a]	0.001
	ethyl-4-hydroxybutyrate#	51.5 [d]	0.7	46.0 [c]	1.7	34.7 [b]	1.0	13.2 [a]	0.6
	ethyl lactate	12.8 [d]	0.1	10.8 [b]	0.0	12.0 [c]	0.1	6.94 [a]	0.02
	monoethyl succinate#	33.5 [b]	0.4	39.2 [c]	0.4	52.5 [d]	0.5	18.7 [a]	0.2
	diethyl succinate	0.691 [a]	0.006	0.964 [c]	0.002	1.640 [d]	0.015	0.867 [b]	0.004
Volatile Fatty Acids	isobutyric acid	1.20 [d]	0.00	1.07 [c]	0.02	1.00 [b]	0.02	0.579 [a]	0.013
	butyric acid	2.59 [b]	0.02	2.55 [b]	0.04	2.54 [b]	0.03	1.33 [a]	0.02
	isovaleric acid	0.808 [d]	0.006	0.777 [c]	0.010	0.746 [b]	0.009	0.412 [a]	0.006
	hexanoic acid	4.84 [c]	0.04	4.47 [b]	0.02	4.88 [c]	0.06	2.30 [a]	0.01
	octanoic acid	6.20 [c]	0.10	5.83 [b]	0.03	6.87 [d]	0.06	3.17 [a]	0.01
	decanoic acid	2.13 [c]	0.02	1.98 [b]	0.01	2.11 [c]	0.02	0.940 [a]	0.070
	lauric acid	0.134 [c]	0.019	0.128 [bc]	0.006	0.096 [b]	0.003	0.039 [a]	0.002
	trans-2-hexenoic acid#	15.6 [b]	1.9	18.4 [c]	0.6	17.5 [bc]	0.2	10.1 [a]	0.4
Acetates of Higher Alcohols	isoamyl acetate	1.42 [d]	0.01	0.830 [c]	0.001	0.546 [b]	0.009	0.265 [a]	0.001
	hexyl acetate	0.105 [d]	0.002	0.084 [c]	0.000	0.051 [b]	0.002	0.028 [a]	0.002
	2-phenylethyl acetate	0.045 [c]	0.001	0.045 [c]	0.003	0.030 [b]	0.002	0.012 [a]	0.001
Carbonyl Compounds	furfural	0.038 [a]	0.001	0.050 [b]	0.003	0.078 [c]	0.004	0.045 [a]	0.000
	benzaldehyde	0.031 [b]	0.001	0.039 [c]	0.001	0.033 [b]	0.002	0.011 [a]	0.002
	acetoine	2.39 [d]	0.10	1.95 [c]	0.04	1.81 [b]	0.05	1.11 [a]	0.20
Volatile Phenols	4-vinyl-phenol	3.80 [bc]	0.24	3.54 [b]	0.16	4.25 [c]	0.19	1.27 [a]	0.02
	4-vinyl-guaiacol	1.25 [b]	0.11	1.54 [c]	0.04	1.98 [d]	0.05	0.440 [b]	0.010
Others	γ-butyrolactone	1.06 [b]	0.79	1.34 [c]	0.00	1.38 [c]	0.01	0.658 [a]	0.010
	methionol#	24.8 [b]	0.6	25.3 [b]	0.7	25.5 [b]	0.5	11.5 [a]	0.2

Concentrations en mg L^{-1}; * en µg L^{-1}; # as normalized area.
SD: Standard deviation of three replicates.
Different letters in the row indicate significant differences among sampling dates for a given compound at $p < 0.05$.

Table S5. Concentrations of volatile compounds (mean ± standard deviation SD) in Treixadura wine sample 5 from the Ribeiro DO at different times of bottle aging. M6, M12, M18 and M24 indicate 6, 12, 18 and 24 months after bottling.

WINE 5		M6		M12		M18		M24	
		Mean	SD	Mean	SD	Mean	SD	Mean	SD
Terpenes	linalool *	22.2 ^{ab}	2.1	27.3 ^{b}	2.4	34.9 ^{c}	2.7	19.1 ^{a}	1.9
	α-terpineol *	6.25 ^{ab}	0.98	4.61 ^{a}	0.65	17.2 ^{c}	1.34	7.78 ^{b}	0.86
C6 Compounds	1-hexanol	1.16 ^{b}	0.01	1.42 ^{c}	0.01	1.46 ^{c}	0.00	0.878 ^{a}	0.022
	cis-3-hexen-1-ol	0.198 ^{b}	0.030	0.157 ^{b}	0.002	0.159 ^{b}	0.001	0.094 ^{a}	0.003
	trans-3-hexen-1-ol	0.079 ^{b}	0.003	0.094 ^{b}	0.001	0.091 ^{b}	0.005	0.055 ^{a}	0.008
Alcohols	methanol	60.6 ^{b}	2.6	57.7 ^{ab}	2.3	71.3 ^{c}	2.7	53.3 ^{a}	1.6
	1-propanol	10.0 ^{a}	2.0	8.6 ^{a}	0.6	6.9 ^{a}	1.7	8.4 ^{a}	0.6
	isobutanol	24.5 ^{b}	0.2	23.7 ^{b}	0.3	25.2 ^{b}	1.4	12.0 ^{a}	1.2
	1-butanol	0.957 ^{a}	0.041	0.948 ^{a}	0.031	1.01 ^{a}	0.091	0.734 ^{a}	0.027
	isoamyl alcohol	198 ^{bc}	2	189 ^{b}	2	209 ^{c}	8	124 ^{a}	2
	benzyl alcohol	0.097 ^{a}	0.002	0.183^{ab}	0.064	0.206 ^{b}	0.003	0.185^{ab}	0.009
	2-phenylethanol	10.8 ^{c}	0.2	9.50 ^{b}	0.10	11.2 ^{d}	0.0	5.86 ^{a}	0.14
	3-methyl-1-pentanol	0.090 ^{b}	0.003	0.088 ^{b}	0.001	0.084 ^{b}	0.001	0.053 ^{a}	0.000
	3-ethoxy-1-propanol#	14.2 ^{b}	0.2	14.5 ^{b}	0.2	13.8 ^{b}	0.2	8.9 ^{a}	0.6
	1,3-butanediol#	277 ^{c}	5	241 ^{d}	3	179 ^{b}	2	126 ^{a}	3
	2,3-butanediol#	61.6 ^{c}	2.4	60.2 ^{c}	0.4	45.7 ^{b}	0.5	33.8 ^{a}	1.4
Esters	ethyl butyrate	0.491 ^{b}	0.008	0.421 ^{b}	0.029	0.453 ^{b}	0.077	0.275 ^{a}	0.012
	ethyl hexanoate	0.772 ^{c}	0.012	0.675 ^{b}	0.004	0.699 ^{bc}	0.002	0.414 ^{a}	0.060
	ethyl octanoate	1.12 ^{b}	0.02	1.59 ^{c}	0.02	1.16 ^{b}	0.02	0.589 ^{a}	0.043
	ethyl decanoate	0.697 ^{b}	0.008	0.966 ^{c}	0.087	0.739 ^{b}	0.002	0.318 ^{a}	0.006
	ethyl-3-hydroxybutyrate	0.131 ^{b}	0.003	0.127 ^{b}	0.016	0.146 ^{b}	0.006	0.075 ^{a}	0.002
	ethyl-4-hydroxybutyrate#	112.0 ^{d}	5.0	94.9 ^{c}	2.1	62.7 ^{b}	1.3	25.6 ^{a}	1.1
	ethyl lactate	10.7 ^{c}	0.2	9.90 ^{b}	0.10	12.9 ^{d}	0.0	7.50 ^{a}	0.17
	monoethyl succinate#	50.0 ^{b}	1.0	57.3 ^{c}	0.3	87.4 ^{d}	0.4	33.9 ^{a}	0.7
	diethyl succinate	0.782 ^{a}	0.009	1.99 ^{d}	0.01	1.96 ^{c}	0.01	1.10 ^{b}	0.00
Volatile Fatty Acids	isobutyric acid	1.30 ^{d}	0.01	1.18 ^{c}	0.01	1.14 ^{b}	0.01	0.679 ^{a}	0.009
	butyric acid	2.65 ^{b}	0.04	2.53 ^{b}	0.03	2.59 ^{b}	0.04	1.42 ^{a}	0.03
	isovaleric acid	0.789 ^{c}	0.008	0.755 ^{b}	0.009	0.772 ^{bc}	0.007	0.417 ^{a}	0.006
	hexanoic acid	4.88 ^{c}	0.05	4.27 ^{b}	0.03	4.83 ^{c}	0.02	2.29 ^{a}	0.05
	octanoic acid	6.64 ^{c}	0.07	6.03 ^{b}	0.03	7.11 ^{d}	0.02	3.21 ^{a}	0.07
	decanoic acid	2.25 ^{c}	0.03	2.05 ^{b}	0.02	2.19 ^{c}	0.01	0.870 ^{a}	0.020
	lauric acid	0.151 ^{bc}	0.007	0.166 ^{c}	0.009	0.128 ^{b}	0.005	0.046 ^{a}	0.001
	trans-2-hexenoic acid#	31.2 ^{b}	1.6	33.3 ^{c}	0.9	32.7 ^{c}	0.8	19.1 ^{a}	0.5
Acetates of Higher Alcohols	isoamyl acetate	2.48 ^{c}	0.03	1.33 ^{b}	0.33	1.22 ^{b}	0.00	0.664 ^{a}	0.016
	hexyl acetate	0.215 ^{c}	0.002	0.675 ^{d}	0.008	0.125 ^{b}	0.003	0.035 ^{a}	0.000
	2-phenylethyl acetate	0.101 ^{c}	0.001	0.100 ^{c}	0.003	0.076 ^{b}	0.002	0.029 ^{a}	0.001
Carbonyl Compounds	furfural	0.030 ^{a}	0.001	0.040 ^{a}	0.002	0.064 ^{a}	0.044	0.053 ^{a}	0.003
	benzaldehyde	0.009 ^{a}	0.002	0.014 ^{b}	0.002	0.017 ^{b}	0.002	0.008 ^{a}	0.001
	acetoine	3.83 ^{c}	0.01	3.13 ^{b}	0.09	2.97 ^{b}	0.04	1.90 ^{a}	0.07
Volatile Phenols	4-vinyl-phenol	2.74 ^{a}	0.15	5.89 ^{b}	0.38	7.16 ^{c}	0.37	2.06 ^{a}	0.18
	4-vinyl-guaiacol	1.48 ^{b}	0.04	1.76 ^{c}	0.03	2.14 ^{d}	0.04	0.603 ^{a}	0.025
Others	γ-butyrolactone	1.30 ^{c}	0.01	1.19 ^{b}	0.01	1.42 ^{d}	0.01	0.651 ^{a}	0.009
	methionol#	47.5 ^{b}	0.6	50.9 ^{c}	0.5	47.3 ^{b}	1.5	22.2 ^{a}	0.8

Concentrations en mg L^{-1}; * en µg L^{-1}; # as normalized area.
SD: Standard deviation of three replicates.
Different letters in the row indicate significant differences among sampling dates for a given compound at $p < 0.05$.

Table S6. Concentrations of volatile compounds (mean ± standard deviation SD) in Treixadura wine sample 6 from the Ribeiro DO at different times of bottle aging. M6, M12, M18 and M24 indicate 6, 12, 18 and 24 months after bottling.

WINE 6		M6		M12		M18		M24	
		Mean	SD	Mean	SD	Mean	SD	Mean	SD
Terpenes	linalool *	21.6 [b]	1.4	24.1 [b]	1.0	33.4 [c]	1.6	15.1 [a]	0.5
C6 Compounds	1-hexanol	0.700 [c]	0.007	0.622 [b]	0.008	0.699 [c]	0.008	0.407 [a]	0.003
	cis-3-hexen-1-ol	0.081 [b]	0.001	0.075 [b]	0.003	0.088 [c]	0.003	0.045 [a]	0.001
	trans-3-hexen-1-ol	0.052 [b]	0.003	0.058 [b]	0.004	0.062 [b]	0.003	0.034 [a]	0.001
Alcohols	methanol	60.6 [a]	1.3	63.9 [a]	4.1	61.3 [a]	2.2	59.0 [a]	3.1
	1-propanol	15.5 [a]	0.7	18.1 [a]	1.5	25.9 [b]	0.5	18.5 [a]	1.7
	isobutanol	16.0 [ab]	0.4	16.2 [b]	0.4	10.5 [ab]	2.6	13.5 [a]	1.7
	1-butanol	1.42 [b]	0.02	1.47 [b]	0.16	1.60 [b]	0.07	0.877 [a]	0.045
	isoamyl alcohol	170 [b]	6	168 [b]	3	177 [b]	2	145 [a]	5
	2-phenylethanol	10.70 [b]	0.10	8.83 [b]	1.85	11.2 [b]	0.1	4.12 [a]	0.03
	3-methyl-1-pentanol	0.085 [b]	0.004	0.080 [b]	0.001	0.088 [b]	0.004	0.048 [a]	0.003
	3-ethoxy-1-propanol#	47.2 [bc]	0.3	43.8 [b]	1.6	47.8 [c]	0.4	21.5 [a]	0.7
	1,3-butanediol#	328 [d]	6	296 [c]	6	283 [b]	5	111 [a]	1
	2,3-butanediol#	67.9 [c]	0.3	57.5 [b]	0.9	66.8 [c]	1.9	25.0 [a]	0.3
Esters	ethyl butyrate	0.540 [b]	0.013	0.490 [b]	0.142	0.526 [b]	0.029	0.264 [a]	0.054
	ethyl hexanoate	0.661 [b]	0.007	0.208 [a]	0.308	0.611 [b]	0.005	0.318 [a]	0.004
	ethyl octanoate	0.959 [b]	0.008	1.26 [c]	0.027	1.34 [c]	0.07	0.531 [a]	0.006
	ethyl decanoate	0.576 [b]	0.005	0.548 [b]	0.035	0.631 [c]	0.008	0.300 [a]	0.003
	ethyl-3-hydroxybutyrate	0.201 [c]	0.001	0.189 [b]	0.004	0.220 [d]	0.002	0.095 [a]	0.002
	ethyl-4-hydroxybutyrate#	119.0 [d]	1.0	93.0 [c]	1.0	70.9 [b]	0.3	20.9 [a]	0.7
	ethyl lactate	8.67 [c]	0.08	7.83 [b]	0.13	10.60 [d]	0.10	4.94 [a]	0.06
	monoethyl succinate#	45.9 [b]	0.4	59.5 [c]	1.1	87.5 [d]	0.5	25.8 [a]	0.6
	diethyl succinate	0.638 [a]	0.006	0.799 [c]	0.013	1.40 [d]	0.02	0.712 [a]	0.007
Volatile Fatty Acids	isobutyric acid	0.893 [c]	0.026	0.799 [b]	0.013	0.876 [c]	0.011	0.413 [a]	0.010
	butyric acid	2.94 [c]	0.02	2.70 [b]	0.04	3.09 [d]	0.04	1.35 [a]	0.02
	isovaleric acid	0.772 [c]	0.006	0.726 [b]	0.019	0.820 [d]	0.013	0.375 [a]	0.009
	hexanoic acid	4.50 [c]	0.04	4.00 [b]	0.10	4.54 [c]	0.05	1.96 [a]	0.02
	octanoic acid	6.32 [c]	0.05	5.92 [b]	0.07	6.85 [d]	0.08	3.29 [a]	0.03
	decanoic acid	1.91 [bc]	0.02	1.87 [b]	0.03	1.96 [c]	0.02	0.865 [a]	0.006
	lauric acid	0.142 [c]	0.014	0.128 [bc]	0.005	0.111 [b]	0.012	0.052 [a]	0.006
Acetates of Higher Alcohols	isoamyl acetate	3.30 [c]	0.03	1.78 [b]	0.48	1.59 [b]	0.01	0.634 [a]	0.007
	hexyl acetate	0.116 [a]	0.002	0.089 [b]	0.001	0.611 [d]	0.002	0.318 [c]	0.001
	2-phenylethyl acetate	0.178 [d]	0.002	0.147 [c]	0.005	0.130 [b]	0.001	0.043 [a]	0.001
Carbonyl Compounds	furfural	0.034 [a]	0.001	0.042 [a]	0.001	0.097 [c]	0.002	0.052 [b]	0.002
	benzaldehyde	0.037 [b]	0.001	0.044 [b]	0.001	0.043 [b]	0.001	0.029 [a]	0.002
	acetoine	1.90 [c]	0.05	1.61 [b]	0.03	1.8 [c]	0.05	0.830 [a]	0.032
Volatile Phenols	4-vinyl-phenol	2.90 [b]	0.11	6.05 [d]	0.64	4.59 [c]	0.52	1.68 [a]	0.18
	4-vinyl-guaiacol	1.33 [b]	0.01	1.82 [c]	0.02	2.27 [d]	0.03	0.520 [a]	0.013
Others	γ-butyrolactone	1.68 [c]	0.12	1.51 [b]	0.02	1.87 [c]	0.02	0.782 [a]	0.117
	methionol#	36.2 [b]	0.1	35.3 [b]	0.7	43.7 [c]	1.4	20.9 [a]	0.5

Concentrations en mg L^{-1}; * en µg L^{-1}; # as normalized area.
SD: Standard deviation of three replicates.
Different letters in the row indicate significant differences among sampling dates for a given compound at $p < 0.05$.

Table S7. Concentrations of volatile compounds (mean ± standard deviation SD) in Treixadura wine sample 7 from the Ribeiro DO at different times of bottle aging. M6, M12, M18 and M24 indicate 6, 12, 18 and 24 months after bottling.

WINE 7		M6		M12		M18		M24	
		Mean	SD	Mean	SD	Mean	SD	Mean	SD
Terpenes	linalool *	34.9 b	2.9	-	-	45.4 c	3.5	20.8 a	2.1
	α-terpineol *	19.0 b	0.1	-	-	31.8 c	2.7	17.0 a	0.6
C6 Compounds	1-hexanol	1.7 b	0.0	-	-	2.7 c	0.1	0.868 a	0.008
	cis-3-hexen-1-ol	0.123 b	0.001	-	-	0.117 b	0.001	0.067 a	0.004
	trans-3-hexen-1-ol	0.049 b	0.003	-	-	0.062 b	0.002	0.038 a	0.001
Alcohols	methanol	65.3 a	2.6	69.9 ab	3.7	73.1 bc	1.9	77.1 c	1.3
	1-propanol	12.1 a	2.4	10.2 a	0.5	10.7 a	0.6	25.4 b	0.5
	isobutanol	25.8 b	1.2	25.8 b	0.3	26.9 b	0.9	23.4 a	0.3
	1-butanol	0.849 a	0.008	-	-	1.070 b	0.190	0.739 a	0.037
	isoamyl alcohol	128 b	6	126 b	1	130 b	3	116 a	4
	benzyl alcohol	0.118 b	0.008	-	-	0.136 b	0.014	0.086 a	0.001
	2-phenylethanol	6.36 b	0.02	-	-	7.31 c	0.04	2.53 a	0.03
	3-ethoxy-1-propanol#	10.20 c	0.50	-	-	9.34 b	0.16	8.06 a	0.24
	1,2-propanodiol#	4.85 a	0.16	-	-	6.16 a	1.20	3.85 a	0.18
	1,3-butanediol#	264.0 c	2.0	-	-	192.0 b	1.0	72.9 a	2.0
	2,3-butanediol#	64.2 c	0.4	-	-	56.9 b	0.7	22.3 a	0.2
Esters	ethyl butyrate	0.394 b	0.004	-	-	0.425 b	0.026	0.275 a	0.019
	ethyl hexanoate	0.556 b	0.002	-	-	0.575 b	0.010	0.291 a	0.002
	ethyl octanoate	1.97 c	0.01	-	-	1.39 b	0.03	0.667 a	0.004
	ethyl decanoate	0.524 b	0.006	-	-	0.617 c	0.028	0.277 a	0.003
	ethyl-3-hydroxybutyrate	0.129 b	0.000	-	-	0.148 c	0.003	0.070 a	0.002
	ethyl-4-hydroxybutyrate#	201 c	1	-	-	110 b	3	41 a	0
	ethyl lactate	28.0 b	0.2	-	-	37.5 c	0.2	20.8 a	0.3
	monoethyl succinate#	53.7 b	0.0	-	-	81.5 c	0.6	28.0 a	0.2
	diethyl succinate	0.691 a	0.004	-	-	1.52 c	0.01	0.721 b	0.009
Volatile Fatty Acids	isobutyric acid	0.872 c	0.050	-	-	0.826 b	0.011	0.496 a	0.009
	butyric acid	2.53 b	0.01	-	-	2.61 b	0.01	1.40 a	0.02
	isovaleric acid	0.478 b	0.001	-	-	0.516 c	0.005	0.272 a	0.004
	hexanoic acid	4.30 b	0.05	-	-	4.50 c	0.03	1.98 a	0.02
	octanoic acid	6.08 b	0.04	-	-	6.82 c	0.04	2.98 a	0.03
	decanoic acid	2.05 b	0.01	-	-	2.14 c	0.01	0.816 a	0.017
	lauric acid	0.256 c	0.008	-	-	0.154 b	0.011	0.055 a	0.008
	trans-2-hexenoic acid#	20.5 b	1.1	-	-	24.0 c	0.4	11.2 a	0.3
Acetates of Higher Alcohols	isoamyl acetate	1.11 c	0.01	-	-	0.642 b	0.004	0.299 a	0.004
	hexyl acetate	0.106 c	0.001	-	-	0.08 b	0.001	0.037 a	0.002
	2-phenylethyl acetate	0.040 c	0.001	-	-	0.031 b	0.002	0.014 a	0.001
Carbonyl Compounds	furfural	0.016 a	0.004	-	-	0.042 b	0.003	0.022 a	0.001
	benzaldehyde	0.045 b	0.002	-	-	0.052 c	0.002	0.018 a	0.00
	acetoine	8.16 c	0.05	-	-	6.90 b	0.10	4.33 a	0.13
Volatile Phenols	4-vinyl-phenol	5.52 b	0.19	-	-	8.27 c	0.66	2.20 a	0.20
	4-vinyl-guaiacol	1.65 b	0.06	-	-	2.94 c	0.02	0.452 a	0.011
Others	γ-butyrolactone	2.76 b	0.02	-	-	3.06 c	0.03	1.33 a	0.02
	methionol#	27.4 b	0.2	-	-	32.0 c	1.1	14.8 a	0.2

Concentrations en mg L^{-1}; * en µg L^{-1}; # as normalized area.
SD: Standard deviation of three replicates.
Different letters in the row indicate significant differences among sampling dates for a given compound at $p < 0.05$.

Table S8. Concentrations of volatile compounds (mean ± standard deviation SD) in Treixadura wine sample 8 from the Ribeiro DO at different times of bottle aging. M6, M12, M18 and M24 indicate 6, 12, 18 and 24 months after bottling.

WINE 8		M6		M12		M18		M24	
		Mean	SD	Mean	SD	Mean	SD	Mean	SD
Terpenes	linalool *	47.5 ^{c}	3.8	34.9 ^{b}	1.2	32.8 ^{b}	2.2	20.4 ^{a}	1.2
	α-terpineol *	51.0 ^{b}	1.3	50.8 ^{b}	2.2	65.2 ^{c}	0.7	28.7 ^{a}	0.4
C6 Compounds	1-hexanol	1.26 ^{d}	0.03	1.07 ^{b}	0.01	1.14 ^{c}	0.01	0.690 ^{a}	0.020
	cis-3-hexen-1-ol	0.087 ^{b}	0.003	0.073 ^{b}	0.002	0.083 ^{b}	0.000	0.053 ^{a}	0.003
	trans-3-hexen-1-ol	0.091 ^{b}	0.003	0.082 ^{b}	0.005	0.087 ^{b}	0.004	0.060 ^{a}	0.008
Alcohols	methanol	68.4 ^{c}	3.0	60.0 ^{b}	3.0	54.8 ^{b}	4	52.6 ^{a}	2.1
	1-propanol	18.0 ^{b}	1.6	13.6 ^{a}	1.8	13.8 ^{a}	1.7	14.3 ^{ab}	0.4
	isobutanol	21.4 ^{bc}	1.3	19.6 ^{ab}	0.6	23.6 ^{c}	1.7	18.3 ^{a}	0.3
	1-butanol	1.15 ^{c}	0.06	0.985 ^{b}	0.026	1.04 ^{bc}	0.06	0.831 ^{a}	0.041
	isoamyl alcohol	177 ^{b}	2	180 ^{b}	2	180 ^{b}	5	165 ^{a}	4
	2-phenylethanol	0.084 ^{b}	0.005	0.970 ^{d}	0.007	0.199 ^{c}	0.009	0.059 ^{a}	0.004
	3-methyl-1-pentanol	8.92 ^{c}	0.27	7.63 ^{b}	0.07	9.03 ^{c}	0.05	3.94 ^{a}	0.06
	3-ethoxy-1-propanol#	39.9 ^{d}	1.2	32.8 ^{b}	0.5	35.2 ^{c}	0.1	21.9 ^{a}	0.5
	1,3-butanediol#	307 ^{d}	3	229 ^{b}	3	242 ^{c}	1	117 ^{a}	5
	2,3-butanediol#	71.4 ^{d}	1.4	52.5 ^{b}	0.7	55.9 ^{c}	0.4	25.1 ^{a}	0.6
Esters	ethyl butyrate	0.468 ^{b}	0.007	0.451 ^{b}	0.042	0.457 ^{b}	0.009	0.292 ^{a}	0.022
	ethyl hexanoate	0.708 ^{c}	0.02	0.590 ^{b}	0.004	0.655 ^{c}	0.009	0.351 ^{a}	0.020
	ethyl octanoate	1.34 ^{b}	0.06	1.40 ^{b}	0.00	1.37 ^{b}	0.11	0.693 ^{a}	0.021
	ethyl decanoate	0.684 ^{b}	0.013	0.684 ^{b}	0.004	0.656 ^{b}	0.001	0.287 ^{a}	0.007
	ethyl-3-hydroxybutyrate	0.146 ^{bc}	0.004	0.133 ^{b}	0.003	0.158 ^{c}	0.003	0.082 ^{a}	0.003
	ethyl-4-hydroxybutyrate#	57.6 ^{d}	2.6	45.8 ^{b}	1.2	51.3 ^{c}	0.4	21.5 ^{a}	1.0
	ethyl lactate	10.01 ^{d}	0.20	7.75 ^{b}	0.01	9.34 ^{c}	0.07	5.49 ^{a}	0.07
	monoethyl succinate#	68.9 ^{c}	1.7	62.3 ^{b}	0.5	88.7 ^{d}	0.7	32.5 ^{a}	0.5
	diethyl succinate	1.17 ^{a}	0.02	1.32 ^{c}	0.01	2.38 ^{b}	0.01	1.27 ^{d}	0.02
Volatile Fatty Acids	isobutyric acid	1.38 ^{c}	0.03	1.12 ^{b}	0.01	1.28 ^{c}	0.08	0.723 ^{a}	0.011
	butyric acid	3.06 ^{b}	0.07	2.62 ^{b}	0.03	2.89 ^{b}	0.35	1.64 ^{a}	0.04
	isovaleric acid	1.05 ^{c}	0.03	0.873 ^{b}	0.013	0.993 ^{c}	0.015	0.535 ^{a}	0.023
	hexanoic acid	4.87 ^{c}	0.13	4.27 ^{b}	0.05	4.88 ^{c}	0.03	2.51 ^{a}	0.04
	octanoic acid	8.00 ^{c}	0.20	7.05 ^{b}	0.07	7.19 ^{b}	0.04	3.52 ^{a}	0.06
	decanoic acid	3.06 ^{c}	0.07	2.21 ^{b}	0.02	2.26 ^{b}	0.02	0.950 ^{a}	0.030
	lauric acid	0.035 ^{a}	0.026	0.324 ^{b}	0.019	0.331 ^{b}	0.024	0.401 ^{c}	0.012
	trans-2-hexenoic acid#	14.4 ^{c}	0.2	11.4 ^{b}	0.3	16.1 ^{d}	0.5	8.2 ^{a}	0.2
Acetates of Higher Alcohols	isoamyl acetate	1.17 ^{d}	0.03	0.70 ^{c}	0.01	0.419 ^{b}	0.009	0.201 ^{a}	0.002
	hexyl acetate	0.069 ^{c}	0.013	0.063 ^{bc}	0.003	0.036 ^{a}	0.001	0.037 ^{ab}	0.001
	2-phenylethyl acetate	0.059 ^{c}	0.004	0.052 ^{c}	0.001	0.041 ^{b}	0.002	0.011 ^{a}	0.001
Carbonyl Compounds	furfural	0.035 ^{a}	0.000	0.041 ^{b}	0.001	0.102 ^{d}	0.005	0.067 ^{c}	0.002
	benzaldehyde	0.014 ^{a}	0.000	0.014 ^{a}	0.001	0.015 ^{a}	0.001	0.008 ^{a}	0.001
	acetoine	2.18 ^{c}	0.09	1.66 ^{b}	0.02	1.84 ^{c}	0.02	1.14 ^{a}	0.03
Volatile Phenols	4-vinyl-phenol	5.04 ^{b}	0.33	6.42 ^{c}	0.80	4.92 ^{b}	0.16	2.76 ^{a}	0.02
	4-vinyl-guaiacol	1.38 ^{c}	0.08	1.14 ^{b}	0.01	1.38 ^{c}	0.05	0.325 ^{a}	0.011
Others	γ-butyrolactone	2.42 ^{b}	0.05	1.90 ^{a}	0.02	2.37 ^{b}	0.01	1.97 ^{a}	0.02
	methionol#	39.4 ^{c}	0.5	34.2 ^{b}	0.5	41.2 ^{d}	0.7	12.8 ^{a}	0.6

Concentrations en mg L^{-1}; * en μg L^{-1}; # as normalized area.
SD: Standard deviation of three replicates.
Different letters in the row indicate significant differences among sampling dates for a given compound at $p < 0.05$.

Table S9. Bottling storage effects on the odor activity values of wines from Treixadura in Ribeiro Designation of Origin (Samples 1-4). M6, M12, M18 and M24 indicate 6, 12, 18 and 24 months after bottling.

Compound	Wine 1				Wine 2				Wine 3				Wine 4			
	M6	M12	M18	M24	M6	M12	M18	M24	M6	M12	M18	M24	M6	M12	M18	M24
linalool	0.6	0.6	0.5	0.3	0.6	0.6	0.6	-	0.5	0.6	0.5	0.4	-	-	-	-
α-terpineol	0.0	0.0	0.1	0.0	-	-	-	-	-	-	-	-	-	-	-	-
1-hexanol	0.3	0.3	0.3	0.2	0.4	0.3	0.4	-	0.3	0.3	0.3	0.2	0.3	0.3	0.3	0.2
cis-3-hexen-1-ol	0.2	0.1	0.1	0.1	0.1	0.1	0.1	-	0.8	0.6	0.6	0.8	0.1	0.1	0.2	0.1
trans-3-hexen-1-ol	0.0	0.0	0.0	0.0	0.0	0.0	0.0	-	0.1	0.1	0.1	0.0	0.0	0.0	0.0	0.0
methanol	0.0	0.0	0.0	0.0	0.0	0.0	0.0	0.0	0.0	0.0	0.0	0.0	0.0	0.0	0.0	0.0
1-propanol	0.5	0.4	0.4	0.5	0.7	0.6	0.5	0.7	0.7	0.4	0.5	0.5	0.3	0.3	0.3	0.4
isobutanol	0.2	0.2	0.4	0.2	0.3	0.2	0.2	0.2	0.3	0.3	0.3	0.3	0.2	0.2	0.4	0.3
1-butanol	0.1	0.2	0.1	0.1	0.1	0.1	0.1	-	0.2	0.2	0.1	0.1	0.1	0.1	0.1	0.1
isoamyl alcohol	4.0	3.9	3.3	4.0	4.8	4.4	4.1	4.3	4.1	3.6	3.9	3.8	3.2	3.2	3.3	4.6
benzyl alcohol	0.0	0.0	0.0	0.0	0.0	0.0	0.0	-	0.0	0.0	0.0	0.0	0.0	0.0	0.0	0.0
2-phenylethanol	0.6	0.5	0.6	0.3	0.7	0.6	0.8	-	0.6	0.5	0.6	0.2	0.4	0.4	0.4	0.2
ethyl butyrate	0.2	0.5	1.6	1.1	1.6	1.2	0.6	-	1.3	1.1	0.9	0.8	1.0	0.6	1.0	0.7
ethyl hexanoate	10.0	9.3	9.2	5.3	6.0	5.4	8.2	-	6.9	6.1	5.8	3.3	8.8	7.9	7.7	4.6
ethyl octanoate	2.2	3.1	2.3	1.3	3.2	2.8	1.6	-	3.3	2.2	1.9	0.9	1.6	2.4	1.8	1.1
ethyl decanoate	1.2	1.0	1.4	0.7	1.1	1.3	1.2	-	0.9	0.9	1.1	0.5	1.2	1.2	1.2	0.6
ethyl lactate	0.1	0.1	0.1	0.0	0.1	0.1	0.1	-	0.1	0.1	0.1	0.0	0.1	0.1	0.1	0.0
diethyl succinate	0.5	0.7	1.2	0.3	0.9	1.1	1.9	-	0.5	0.8	1.4	0.8	0.6	0.8	1.4	0.7
isobutyric acid	0.3	0.3	0.3	0.2	0.3	0.3	0.3	-	0.5	0.4	0.4	0.2	0.5	0.5	0.4	0.3
butyric acid	0.8	0.8	0.8	0.7	1.0	0.8	0.9	-	0.6	0.6	0.6	0.3	0.6	0.6	0.6	0.3
isovaleric acid	23.0	22.4	23.8	13.7	24.7	21.6	22.9	-	23.6	22.4	22.4	12.6	26.9	25.9	24.9	13.7
hexanoic acid	1.8	1.7	1.8	0.8	1.7	1.4	1.7	-	1.2	1.1	1.2	0.6	1.6	1.5	1.6	0.8
octanoic acid	0.7	0.7	0.8	0.3	0.5	0.6	0.7	-	0.5	0.5	0.6	0.3	0.6	0.6	0.7	0.3
decanoic acid	0.4	0.3	0.4	0.1	0.3	5.9	6.3	-	0.3	0.3	0.3	0.1	0.4	0.3	0.4	0.2
isoamyl acetate	13.4	9.5	5.7	2.6	15.9	9.8	6.8	-	18.1	12.9	8.4	4.2	8.9	5.2	3.4	1.7
hexyl acetate	0.2	0.1	0.1	0.1	0.3	0.1	0.1	-	0.3	0.1	0.2	0.1	0.2	0.1	0.1	0.0
2-phenylethyl acetate	0.1	0.0	0.0	0.0	0.1	0.1	0.0	-	0.1	0.1	0.1	0.0	0.0	0.0	0.0	0.0
furfural	0.0	0.0	0.0	0.0	0.0	0.0	0.0	-	0.0	0.0	0.0	0.0	0.0	0.0	0.0	0.0
benzaldehyde	0.0	0.0	0.0	0.0	0.0	0.0	0.0	-	0.0	0.0	0.0	0.0	0.0	0.0	0.0	0.0
acetoine	0.0	0.0	0.0	0.0	0.0	0.0	0.0	-	0.0	0.0	0.0	0.0	0.0	0.0	0.0	0.0
4-vinyl-phenol	6.7	8.8	15.3	5.2	9.7	20.2	16.1	-	12.1	15.8	12.7	4.2	10.1	9.4	11.3	3.4
4-vinyl-guaiacol	2.7	3.9	4.8	1.1	3.9	4.0	3.8	-	2.5	3.0	3.9	1.1	2.8	3.5	4.5	1.0
0.2 > OAV > 1	13	12	11	11	10	8	9	-	14	13	12	14	10	10	9	12
OAV > 1	9	9	9	8	11	12	11	-	9	9	10	6	10	9	11	7
TOTAL	**22**	**21**	**20**	**19**	**21**	**20**	**20**	**-**	**23**	**22**	**22**	**20**	**20**	**19**	**20**	**19**

Table S10. Bottling storage effects on the odor activity values of wines from Treixadura in Ribeiro Designation of Origin (Samples 5-8). M6, M12, M18 and M24 indicate 6, 12, 18 and 24 months after bottling.

Compound	Wine 5				Wine 6				Wine 7				Wine 8			
	**M6**	**M12**	**M18**	**M24**	**M6**	**M12**	**M18**	**M24**	**M6**	**M12**	**M18**	**M24**	**M6**	**M12**	**M18**	**M24**
linalool	0.4	0.5	0.7	0.4	0.4	0.5	0.7	0.3	0.7	-	0.9	0.4	1.0	0.7	0.7	0.4
α-terpineol	0.0	0.0	0.0	0.0	-	-	-	-	0.0	-	0.1	0.0	0.1	0.1	0.2	0.1
1-hexanol	0.3	0.4	0.4	0.2	0.2	0.2	0.2	0.1	0.4	-	0.7	0.2	0.3	0.3	0.3	0.2
cis-3-hexen-1-ol	0.2	0.2	0.2	0.1	0.1	0.1	0.1	0.0	0.1	-	0.1	0.1	0.1	0.1	0.1	0.1
trans-3-hexen-1-ol	0.0	0.0	0.0	0.0	0.0	0.0	0.0	0.0	0.0	-	0.0	0.0	0.0	0.0	0.0	0.0
methanol	0.0	0.0	0.0	0.0	0.0	0.0	0.0	0.0	0.0	0.0	0.0	0.0	0.0	0.0	0.0	0.0
1-propanol	0.3	0.3	0.2	0.3	0.5	0.6	0.9	0.6	0.4	0.3	0.4	0.8	0.6	0.5	0.5	0.5
isobutanol	0.3	0.3	0.3	0.2	0.2	0.2	0.1	0.2	0.3	0.3	0.4	0.3	0.3	0.3	0.3	0.2
1-butanol	0.1	0.1	0.1	0.1	0.1	0.1	0.1	0.1	0.1	-	0.1	0.1	0.1	0.1	0.1	0.1
isoamyl alcohol	5.0	4.7	5.2	3.1	4.3	4.2	4.4	3.6	3.2	3.2	3.3	2.9	4.4	4.5	4.5	4.1
benzyl alcohol	0.0	0.0	0.0	0.0	0.0	0.0	0.0	0.0	0.0	-	0.0	0.0	0.0	0.0	0.0	0.0
2-phenylethanol	0.8	0.7	0.8	0.4	0.8	0.6	0.8	0.3	0.5	-	0.5	0.2	0.6	0.5	0.6	0.3
ethyl butyrate	1.2	1.1	1.1	1.1	1.4	1.2	1.3	0.7	1.0	-	1.1	0.7	1.2	1.1	1.1	0.7
ethyl hexanoate	9.7	8.4	8.7	8.7	8.3	2.6	7.6	4.0	7.0	-	7.2	3.6	8.9	7.4	8.2	4.4
ethyl octanoate	1.9	2.7	2.0	2.0	1.7	2.2	2.3	0.9	3.4	-	2.4	1.2	2.3	2.4	2.4	1.2
ethyl decanoate	1.4	1.9	1.5	1.5	1.2	1.1	1.3	0.6	1.0	-	1.2	0.6	1.4	1.4	1.3	0.6
ethyl lactate	0.1	0.1	0.1	0.1	0.1	0.1	0.1	0.0	0.2	-	0.3	0.1	0.1	0.1	0.1	0.0
diethyl succinate	0.7	1.7	1.6	0.9	0.5	0.7	1.2	0.6	0.6	-	1.3	0.6	1.0	1.1	2.0	1.1
isobutyric acid	0.6	0.5	0.5	0.3	0.4	0.3	0.4	0.2	0.4	-	0.4	0.2	0.6	0.5	0.6	0.3
butyric acid	0.7	0.6	0.6	0.4	0.7	0.7	0.8	0.3	0.6	-	0.7	0.4	0.8	0.7	0.7	0.4
isovaleric acid	26.3	25.2	25.7	13.9	25.7	24.2	27.3	12.5	15.9	-	17.2	9.1	35.0	29.1	33.1	17.8
hexanoic acid	1.6	1.4	1.6	0.8	1.5	1.3	1.5	0.7	1.4	-	1.5	0.7	1.6	1.4	1.6	0.8
octanoic acid	0.7	0.6	0.7	0.3	0.6	0.6	0.7	0.3	0.6	-	0.7	0.3	0.8	0.7	0.7	0.4
decanoic acid	0.4	0.3	0.4	0.1	0.3	0.3	0.3	0.1	0.3	-	0.4	0.1	0.5	0.4	0.4	0.2
isoamyl acetate	15.5	8.3	7.6	4.2	20.6	11.1	9.9	4.0	6.9	-	4.0	1.9	7.3	4.4	2.6	1.3
hexyl acetate	0.3	1.0	0.2	0.1	0.2	0.1	0.9	0.5	0.2	-	0.1	0.1	0.1	0.1	0.1	0.1
2-phenylethyl acetate	0.1	0.1	0.0	0.0	0.1	0.1	0.1	0.0	0.0	-	0.0	0.0	0.0	0.0	0.0	0.0
furfural	0.0	0.0	0.0	0.0	0.0	0.0	0.0	0.0	0.0	-	0.0	0.0	0.0	0.0	0.0	0.0
benzaldehyde	0.0	0.0	0.0	0.0	0.0	0.0	0.0	0.0	0.0	-	0.0	0.0	0.0	0.0	0.0	0.0
acetoine	0.0	0.0	0.0	0.0	0.0	0.0	0.0	0.0	0.1	-	0.0	0.0	0.0	0.0	0.0	0.0
4-vinyl-phenol	7.3	15.7	19.1	5.5	7.7	16.1	12.2	4.5	14.7	-	22.1	5.9	13.4	17.1	13.1	7.4
4-vinyl-guaiacol	3.4	4.0	4.9	1.4	3.0	4.1	5.2	1.2	3.8	-	6.7	1.0	3.1	2.6	3.1	0.7
0,2 > OAV > 1	12	10	11	9	10	10	9	13	11	-	10	12	8	9	10	13
OAV > 1	10	12	11	10	10	10	11	6	10	-	11	7	12	11	11	7
TOTAL	**22**	**22**	**22**	**19**	**20**	**20**	**20**	**19**	**21**	**-**	**21**	**19**	**20**	**20**	**21**	**20**
